# Targeting WWP1 ameliorates cardiac ischemic injury by suppressing KLF15-ubiquitination mediated myocardial inflammation

**DOI:** 10.7150/thno.77694

**Published:** 2023-01-01

**Authors:** Xia Lu, Boshen Yang, Ruiqiang Qi, Qifei Xie, Taixi Li, Jie Yang, Tingting Tong, Kaifan Niu, mingyu Li, Weijun Pan, Yongxin Zhang, Dongmei Shi, Suiji Li, Cuilian Dai, Chengxing Shen, Xiaoqing Wang, Yan Wang, Juan Song

**Affiliations:** 1 Department of Cardiology, Shanghai Sixth People's Hospital Affiliated to Shanghai Jiao Tong University School of Medicine, Shanghai 200233, China.; 2 Xiamen Cardiovascular Hospital, Xiamen University, Xiamen 361004, China.; 3 Key Laboratory of Targeted Intervention of Cardiovascular Disease, Collaborative Innovation Center for Cardiovascular Disease Translational Medicine, Nanjing Medical University, Nanjing 211166, Jiangsu, China.; 4 Fujian Provincial Key Laboratory of Innovative Drug Target Research, School of Pharmaceutical Sciences, Xiamen University, Xiamen 361102, China.; 5 Key Laboratory of Tissue Microenvironment and Tumor, CAS Center for Excellence in Molecular Cell Science, Shanghai Institute of Nutrition and Health, Shanghai Institutes for Biological Sciences, University of Chinese Academy of Sciences, Chinese Academy of Sciences (CAS), Shanghai, China.; 6 The first clinical medical college, Southern Medical University, Guangzhou 510000, China.

**Keywords:** myocardial infarction, cardiomyocyte inflammation, WWP1, KLF15-Ubiquitination

## Abstract

**Rationale:** Previous studies have suggested that myocardial inflammation plays a critical role after ischemic myocardial infarction (MI); however, the underlying mechanisms still need to be fully elucidated. WW domain-containing ubiquitin E3 ligase 1 (WWP1) is considered as an important therapeutic target for cardiovascular diseases due to its crucial function in non-ischemic cardiomyopathy, though it remains unknown whether targeting WWP1 can alleviate myocardial inflammation and ischemic injury post-MI.

**Methods:** Recombinant adeno-associated virus 9 (rAAV9)-cTnT-mediated WWP1 or Kruppel-like factor 15 (KLF15) gene transfer and a natural WWP1 inhibitor Indole-3-carbinol (I3C) were used to determine the WWP1 function in cardiomyocytes. Cardiac function, tissue injury, myocardial inflammation, and signaling changes in the left ventricular tissues were analyzed after MI. The mechanisms underlying WWP1 regulation of cardiomyocyte phenotypes* in vitro* were determined using the adenovirus system.

**Results:** We found that WWP1 expression was up-regulated in cardiomyocytes located in the infarct border at the early phase of MI and in hypoxia-treated neonatal rat cardiac myocytes (NRCMs). Cardiomyocyte-specific WWP1 overexpression augmented cardiomyocyte apoptosis, increased infarct size and deteriorated cardiac function. In contrast, inhibition of WWP1 in cardiomyocytes mitigated MI-induced cardiac ischemic injury. Mechanistically, WWP1 triggered excessive cardiomyocyte inflammation after MI by targeting KLF15 to catalyze K48-linked polyubiquitination and degradation. Ultimately, WWP1-mediated degradation of KLF15 contributed to the up-regulation of p65 acetylation, and activated the inflammatory signaling of MAPK in ischemic myocardium and hypoxia-treated cardiomyocytes. Thus, targeting of WWP1 by I3C protected against cardiac dysfunction and remodeling after MI.

**Conclusions:** Our study provides new insights into the previously unrecognized role of WWP1 in cardiomyocyte inflammation and progression of ischemic injury induced by MI. Our findings afford new therapeutic options for patients with ischemic cardiomyopathy.

## Introduction

Myocardial infarction (MI), is associated with increased morbidity and mortality and is defined as sudden ischemic death of myocardial cells 1. The inability of cardiomyocytes to regenerate from cardiac progenitor cells in the mammalian adult heart is a clinically intractable problem 2. Salvaging the cardiomyocytes that are otherwise destined to die remains a pivotal objective in the scientific research of ischemic cardiomyopathy.

Molecular mechanisms modulating the survival of cardiomyocytes and myocardial injury are under investigation. The post-translational modification of protein ubiquitination has been shown to be closely related to cardiovascular diseases 3, 4. E3 ubiquitin ligase mediates proteasome degradation by catalyzing the covalent binding of ubiquitin to the substrates 5. The variations in the expression and activities of E3 ubiquitin ligases in the post-ischemic heart milieu influence the fate of cardiomyocytes 6. However, there are no therapeutic approaches targeting the ubiquitin-proteasome system (UPS), suggesting that a more comprehensive understanding of E3 ubiquitin ligase in controlling cardiomyocyte fate is urgently needed to reveal effective therapeutic targets for myocardial infarction.

The WW domain-containing ubiquitin E3 ligase 1 (WWP1), identified as an E3 ubiquitin ligase, contains an N-terminal C2 domain and has been implicated in several diseases, such as cancers, neurological disorders, infectious diseases, and aging 7-9. Seminal studies have demonstrated that WWP1 regulates critical protein turnover and stability, inducing ventricular hypertrophy, diastolic dysfunction, and extracellular matrix deposition 10. Cardiomyocyte-specific overexpression of WWP1 contributes to gap junction remodeling and arrhythmogenesis 11, while gene deletion of WWP1 attenuates diastolic dysfunction in pressure overload-induced pathological cardiac hypertrophy 12. In addition, WWP1 is a potential therapeutic target for cardiac remodeling and functional decline induced by simulated microgravity 13. However, previous studies involving WWP1 focused on non-ischemic heart diseases, and it remains unknown whether WWP1 could regulate cardiac dysfunction and infarct size after acute myocardial ischemic injury.

Inflammation is a critical trigger in response to myocardial injury and cardiomyocyte loss 14, 15. Although resident and infiltrating immune cells, myocardial fibroblasts, and endothelial cells are vital sources of inflammatory mediators, cardiomyocytes can also synthesize and secrete inflammatory factors, which contribute to cardiac pathology 16. In this context, WWP1, an important negative regulator of TLR4-mediated TNF-α and IL-6 production, binds to TRAF6 and promotes K48-linked polyubiquitination 17. WWP1 targets JunB for ubiquitination and degradation to promote inflammation-mediated osteoporosis 18; and regulates Cav3.2 channel ubiquitination for its degradation to enhance neuropathic and inflammatory pain 19. Notably, WWP1 has been reported to be highly expressed in cardiomyocytes, but not in cardiac fibroblasts of adult mice subjected to transverse aortic constriction 20, suggesting that WWP1 is crucial for the pathophysiology of cardiomyocytes (eg, ischemia-induced inflammatory response). Therefore, this study aimed to determine whether and how cardiomyocyte WWP1 elicits an inflammatory response in the post-infarction heart.

Krüppel-like factor 15 (KLF15), a member of the zinc-finger family of transcription factors, controls the survival and function of cardiomyocytes 21, and inhibits acetylation-mediated NF-κB activation through KLF15-p300 interaction 22. Transcriptional activation of NF-κB is a key factor in inducing myocardial inflammation 23. Moreover, overexpression of KLF15 alleviates cardiac dysfunction after myocardial infarction by inhibiting the P38/MAPK signaling pathway 24. Therefore, stimulation or stabilization of KLF15 expression could be crucial for mitigating NF-κB and MAPK activation pathway- mediated cardiomyocyte inflammatory response and injury post-MI. Recently, regulation of polyubiquitination and abundance of KLF15 in hyperglycemia-induced skeletal muscle atrophy by WWP1 has been demonstrated 25. Forced expression of WWP1 reduced the amount and augmented polyubiquitination of KLF15, whereas expression of a catalytically inactive mutant of WWP1 did not influence the ubiquitination modification of KLF15 25. However, the role of WWP1-mediated-ubiquitination and degradation of KLF15 in controlling NF-κB and MAPK activation has not been investigated, which could be the key mechanism of cardiomyocyte inflammatory response.

The primary objective of this study was to demonstrate whether targeting WWP1 could control the inflammatory response of cardiomyocytes in the post-ischemic heart. We also aimed to elucidate the underlying mechanisms that WWP1 promoted KLF15 degradation through ubiquitination and subsequently resulted in NF-κB and MAPK activation. Our study, for the first time, provides evidence that WWP1 is highly expressed in cardiomyocytes and regulates a pro-inflammatory program to aggravate myocardium ischemic injury, and identifies potential therapeutic targets for reducing inflammatory damage in the ischemic heart.

## Materials and Methods

### Animals and surgical procedures

All animal experiments were reviewed and approved by the guidelines for the Care and Use of Laboratory Animals published by the US National Institutes of Health (NIH Publication, 8th Edition, 2011). The animal care and experimental protocols were approved by the intramural Ethics Committee on Humane Treatment of Experimental Animals (Protocol No. XMULAC20160089 of March 10, 2016). The MI mouse model was induced by ligation of the left anterior descending (LAD) coronary artery with a 6-0 prolene suture. 8-week-old male mice anesthetized using a mixture of isoflurane (1.5%) and oxygen (0.5 L/min) were placed on a temperature-controlled surgical table and subjected to mechanical ventilation before the surgical procedure. Afterwards, the thorax was closed and the animals were extubated before they were allowed to recover from the surgery. In sham-operated mice, the same procedure was performed except for LAD occlusion. Burprenox (0.36 mg/kg, i.m.) was employed as analgesia post-operative. Mice were sacrificed on the corresponding days after MI surgery. The animals were euthanized by a mixture of isoflurane and oxygen to obtain their heart samples for corresponding analyses.

### Infarct size measurement

Triphenyltetrazolium chloride (TTC) staining was used to assess the infarct size. The degree of infarct was presented as the percentage of infarct over the ventricular area.

### Echocardiography

Cardiac functions were evaluated by echocardiography (GE Vivid 7 equipped with a 14-MHz phase array linear transducer, S12, allowing a 150 maximal sweep rate) in Figure 2, Figure 8, Figure 11, and Figure S5. Cardiac functions were tested with a high-resolution ultrasound imaging system (MyLab Touch: Esaote, Italy linear array probe, frequency 18-22 MHz) in Figure 7. Detailed information was described in Supplementary materials.

### Cell culture

Neonatal rat cardiac myocytes (NRCMs) were isolated from 1 to 3-day-old neonatal Sprague-Dawley rats (Beijing Vital River Laboratory Animal Technology Co., Ltd.), and detailed information was described in Supplementary materials.

### Western blot

Protein extraction from the mouse heart tissue and cultured cardiomyocytes, respectively. Immunoblots were performed as previously described 4, and detailed information was described in Supplementary materials.

### Immunoprecipitation/Co-immunoprecipitation

Cell and tissue lysates (800 μg) were prepared and incubated with anti-KLF15 (Santa Cruz, 1 µg per 250 µg of total protein) antibody overnight at 4 °C followed by the addition of 30 μL of protein G Agarose beads (cytiva) for 4 h at 4 °C. After being washed three times with cold wash buffer and once with lysis buffer (10 mM HEPES pH = 7.9, 10 mM KCl, 1.5 mM MgCl_2_, 50 mM NaF 1 mM Na3VO4, 1 μM PMSF plus a protease inhibitor cocktail), the immunoprecipitations were resuspended in 30 μL of loading buffer and subjected to western blot for examination of Ub (K48) modification. Cell lysates (> 500 μg) were prepared and incubated with anti-p300 (CST, 1:50) antibody overnight at 4 °C followed by addition of 30 μL of protein G Agarose beads, and the other steps were the same as above. Western blot was performed for examination of interaction between p300 and p65.

### Real-time PCR

Total RNA was extracted, and then converted to cDNA using PrimeScript^TM^ RT Reagent kit with gDNA eraser (Takara RR047A, Japan). Quantitative RT-PCR reactions were performed using ChamQTM SYBR Color qPCR Master Mix (Vazyme, China) and LightCycler^®^ 96 Instrument (Roche, USA). Hypoxanthine-guanine phosphoribosyltransferase (HPRT) was used as an internal reference for each sample. Primer sequences are shown in Supplementary Table.

### Immunofluorescence

The heart tissue chips are dewaxed and rehydrated. Citric acid sodium buffer solution was added for antigen retrieval. For cultured cells, they were washed with phosphate buffered saline (PBS) and then fixed with 4% paraformaldehyde. The washed heart tissue chips or cells were then permeabilized with 0.1% Triton X-100 and blocked with 3% bovine serum. They were stained with one or more corresponding antibodies (cTnI, abcam, 1:100; WWP1, abcam, 1:100; F4/80, abcam, 1:100; KLF15, Santa Cruz, 1:25, DAPI, Invitrogen). Five fields were randomly selected from the infarct, border, and remote zones of MI heart. The border zone was defined as the immediate neighboring area around the infarct. Fluorescence measurements were performed using the confocal microscope (Zeiss, German). The Image J software was applied for quantitative analysis.

### TUNEL assay

The heart tissues were embedded in paraffin, cut at 8 μm, dewaxed and rehydrated, and then permeabilized with 20 μg/mL proteinase K for 10 min. The staining was performed using an *In situ* Cell Death Detection Kit (12156792910, Roche) according to the protocol. Detailed information was described in Supplementary materials.

### Transwell assay

A 24-well transwell chamber with 8 μm pores (Corning, NY) was used to explore the WWP1-mediated effect of inflammatory cytokines released from cardiomyocytes on macrophage chemotaxis. 2.5×10^5^ RAW264 cells in 300 μL serum-free DMEM high glucose medium were seeded in the upper wells. 700 μL conditioned medium from hypoxia or normoxia-treated NRCMs infected with Adv-WWP1 or Adv-GFP were added to the lower wells. After incubation for 24 h, RAW264 cells attached to the lower surface of membrane were fixed with 4% paraformaldehyde for 30 min, and then stained with 0.05% crystal violet. Five fields of vision were randomly selected to be photographed in bright field by microscope (OLYMPUS TH4-200, Japan). The number of cells per field was counted by Image J software.

### Plasmids and transfection

Mouse expression plasmids encoding His-tagged ubiquitin, GFP-tagged KLF15, Flag-tagged WWP1, and HA-tagged WWP1 (C886A), in which Cystein-886 (C886A) was replaced by alanine, were purchased from Genechem (Shanghai, China). For transient transfection, H9C2 or 293T were transfected with the indicated plasmids by NEOFECT^TM^ DNA transfection reagent according to manufacturer's recommendations.

Si-WWP1 used for transfection *in vitro* was obtained from RIBOBIO (Guang zhou, China); Si- WWP1_001: GCTTGGATCTACCACCATA; Si-W WWP1_002: CAGAGGGATTTGGACAAGA; Si-W WWP1_003: GGGATTTGGACAAGAATGA. H9C2 cells were transfected with Si-WWP1 via riboFECT^TM^CP Tansfection Kit according to manufacturer's recommendations.

### Construction of recombinant adenovirus and adeno-associated virus

Recombinant adenovirus (Adv) and adeno-associated virus 9 (rAAV9) packaged by Genechem (Shanghai, China) were used to manipulate the expression of WWP1 in cultured cardiomyocytes and mice hearts, respectively. A heart specific chicken cardiac troponin T (cTnT) promoter was employed to express or suppress the expression of WWP1 or KLF15 in cardiomyocytes. Gene sequence of WWP1/KLF15 was referred to Genbank (NM_177327/NM_023184). A short hairpin RNA (shRNA) was synthesized, which contained a target sequence for WWP1 or KLF15 as follows: TGACTTGAGGAGGCGATTATA (shWWP1); CACCTTCATCCAGAGTCTGCT (shKLF15). The sequence of scramble as follows: TTCTCCGAACGTGTCACGT. Six-week-old wild type male mice (C57BL/6J) were intravenously injected with adeno-associated virus (3×10^11^ v.g./mouse) for two weeks before MI surgery.

### Indole-3-Carbinol (I3C) treatment

I3C was diluted in 5% DMSO and 95% PBS with a final dose of 20 mg/kg. C57BL/6J mice, at 6 weeks of age, were treated with PBS containing 5% DMSO or I3C three times a week for one month via intraperitoneal injection. Cells were treated at the final concentration of 50 μM for 24 h.

### Statistical analysis

Data are displayed as means ± SD. Statistical analysis was carried out using GraphPad Prism 7.0. Comparisons between two groups were made by two-tailed unpaired Student's t-test. For comparison of more than two groups, one-way or two-way analysis of variance (ANOVA) corrected by Bonferroni post hoc test was performed, when the assumption was satisfied (normal distribution). Otherwise, the nonparametric Kruskal-Wallis test was used for further analysis. Differences were considered statistically significant at *p* < 0.05.

## Results

### WWP1 is highly expressed in cardiomyocytes in response to MI

**Figure 1:**
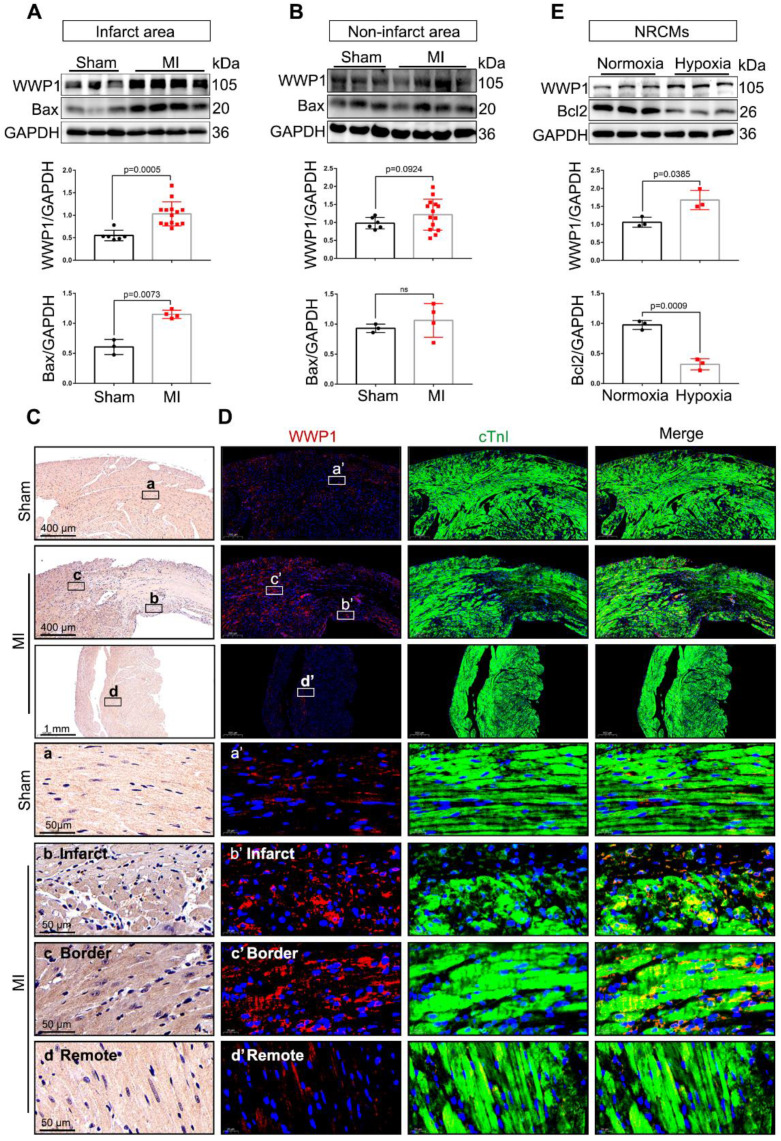
** WWP1 expression is increased in cardiomyocytes in response to myocardial infarction. A** Immunoblot analysis of WWP1 (n = 6, 14) and Bax (n = 3, 4) expression in the infarct areas (infarct and border zone) of wild type (WT) mice at day 1 post-MI as well as in the sham control. Corresponding statistics of WWP1 and Bax were shown. **B** Immunoblot analysis of WWP1 (n = 6, 14) and Bax (n = 3, 4) expressions in non-infarct areas at day 1 post-MI as well as in the sham control. Corresponding statistics of WWP1 and Bax were shown. **C** Immunohistochemistry for WWP1 (brown) in the sham heart or infarcted heart tissue. n = 4.** D** Immunofluorescence co-staining for cTnI with WWP1 and DAPI in the heart post-MI. n = 4. **E** Neonatal rat cardiac myocytes (NRCMs) were treated by hypoxia for 6h. WWP1 and Bcl2 expressions were examined by Western blots. Corresponding statistics of WWP1 and Bcl2 were shown. n = 3. The data are shown as the means ± SD. The data shown in A, B, and E were analysed by unpaired Student's t-test.

We explore the possible involvement of WWP1 in myocardial injury post-MI, by examining the temporal protein expression pattern of WWP1 in the infarct area after MI. Immunoblotting showed increased WWP1 expression that peaked on the first day post-MI (Figure S1). Subsequently, WWP1 expression changes in different zones were evaluated at day 1. Figure 1A shows a dramatical elevation in WWP1 protein level, accompanied by increased expression of pro-apoptotic protein Bax in the infarct area, and border areas at day 1 post-MI (Figure 1A); no detectable change in WWP1 expression was observed in the non-infarct area (Figure 1B). Immunohistochemistry also revealed significantly higher WWP1 protein expression in the infarct and border myocardium than in sham controls (Figure 1C). Also, immunofluorescence staining of heart tissue sections with anti-WWP1 and anti-cTnI antibodies showed strong positive WWP1 staining in cardiomyocytes in the infarct border, but sporadically observed in the remote areas at day 1 post-MI (Figure 1D). Additionally, *in vitro* experiments also demonstrated abundant WWP1 protein expression, accompanied by low anti-apoptotic protein Bcl2 in neonatal rat cardiac myocytes (NRCMs) exposed to simulated ischemia by deprivation of glucose and oxygen (Figure 1E). These data indicated up-regulation of WWP1 in cardiomyocytes located in the infarct border during acute injury progression after MI.

### WWP1 is a driver of cardiac dysfunction post-MI

**Figure 2:**
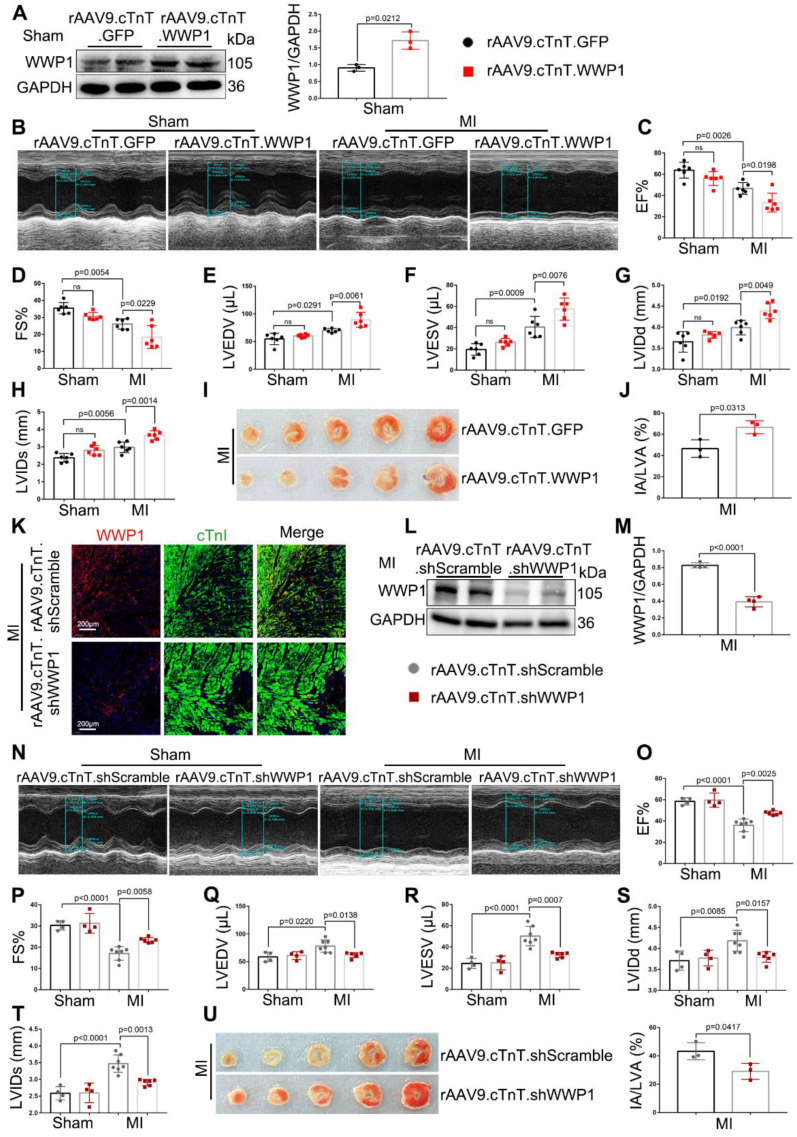
** WWP1 is a driver of cardiac dysfunction and myocardium infarct post-MI.** WT mice were treated with rAAV9-cTnT-GFP or rAAV9-cTnT-WWP1 by intravenous injection of tail two weeks before suffering from sham or LAD ligation, and after additional one day, mice were sacrificed. **A** Infection efficiency was detected by Western blot. n = 3.** B-H** Cardiac function indices were measured by echocardiography. EF% ejection fraction, FS% fractional shortening, LVEDV left ventricular end-diastolic volume, LVESV left ventricular end-systolic volume, LVIDd left ventricular internal dimension diastole, LVIDs left ventricular internal dimension systole. n = 6. **I, J** Infarct size was determined by TTC staining and expressed as the percentage of infarct over ventricular areas. n = 3. **K-U** Mice were treated with rAAV9-cTnT-shScramble or rAAV9-cTnT-shWWP1 by intravenous injection of tail 2 weeks before suffering from sham or LAD ligation, and after additional 3 days, mice were sacrificed. K Immunofluorescence co-staining for cTnI with WWP1 and DAPI in mice hearts infected with rAAV9-cTnT-shScramble or rAAV9-cTnT-shWWP1 at day 3 post-MI. Scale bar = 200 μm. n = 3. **L,** M WWP1 protein level was detected by Western blots. Corresponding statistic of WWP1 was shown. n = 4. N-T Cardiac function indices were measured by echocardiography. n = 4, 4, 7, 6, respectively. **U** Infarct size was determined by TTC staining and expressed as the percentage of infarct over ventricular areas. n = 3. The data are shown as the means ± SD. The data shown in A, C-H, J, M, O-T, and U were analysed by one-way ANOVA followed by Bonferroni post hoc test.

Next, we examined the functional effects of increased WWP1 on myocardial injury induced by MI. We employed rAAV9-cTnT-WWP1 or rAAV9-cTnT-GFP in wild-type (WT) mice two weeks before they were subjected to permanent LAD ligation for one day to further up-regulate WWP1 expression, specifically in cardiomyocytes. As detected by Western blotting, overexpression of WWP1 was successful in isolated cardiomyocytes from sham mice transfected with rAAV9-cTnT-WWP1, compared to the control group (Figure 2A), and led to deteriorated cardiac function at day 1 post-MI. Echocardiographic parameters, including left ventricular (LV) ejection fraction (EF%) and fractional shortening (FS%), LV end-diastolic volume (LVEDV) and end-systolic volume (LVESV), and LV internal diameter at end diastole (LVIDd) and at end systole (LVIDs) were significantly deteriorated in cardiomyocyte-specific WWP1-overexpressing mice subjected to LAD ligation (Figure 2B-H). Triphenyltetrazolium chloride (TTC) staining used to visualize and evaluate the infarct size, revealed that WWP1 overexpression was associated with a considerably enlarged heart infarct area in MI-mice Figure 2I-J).

The first three days are the acute injury period after myocardial infarction 26. Since up-regulation of WWP1 is detrimental to sustained cardiac function at day 1 post-MI, it is plausible that inhibition of WWP1 expression could alleviate myocardial ischemic injury. To this end, we engineered a viral shRNA vector under the control of cTnT promoter to knockdown WWP1 in cardiomyocytes. Mice were injected with rAAV9-cTnT-shWWP1 or rAAV9-cTnT-shScramble for 2 weeks prior to LAD ligation, and then sacrificed 3 days after LAD ligation. The cardiomyocyte-specific WWP1 knockdown was verfied by the absence of WWP1 protein in cardiomyocytes at day 3 post-MI (Figure 2K-M). As expected, WWP1 knockdown in cardiomyocytes attenuated cardiac function after LAD ligation for 3 days, indicated by an increase in EF% and FS%, and a decrease in LVEDV, LVESV, LVIDd, and LVIDs (Figure 2N-T). No significant differences were observed in control sham-mice between the rAAV9-cTnT-shWWP1 and rAAV9-cTnT-shScramble groups. TTC staining also demonstrated significantly reduced infarct size after WWP1 inhibition in cardiomyocytes (Figure 2U). Collectively, these data revealed that highly expressed WWP1 in cardiomyocytes worsens MI-induced cardiac dysfunction and infarct size. In contrast, cardiomyocyte-specific WWP1 knockdown exerts protective effects on ischemic myocardial injury, indicating a potential role for WWP1 in regulating cardiomyocyte death and myocardial injury in the early phase post-MI.

### WWP1 induces cardiomyocyte apoptosis post-MI

**Figure 3:**
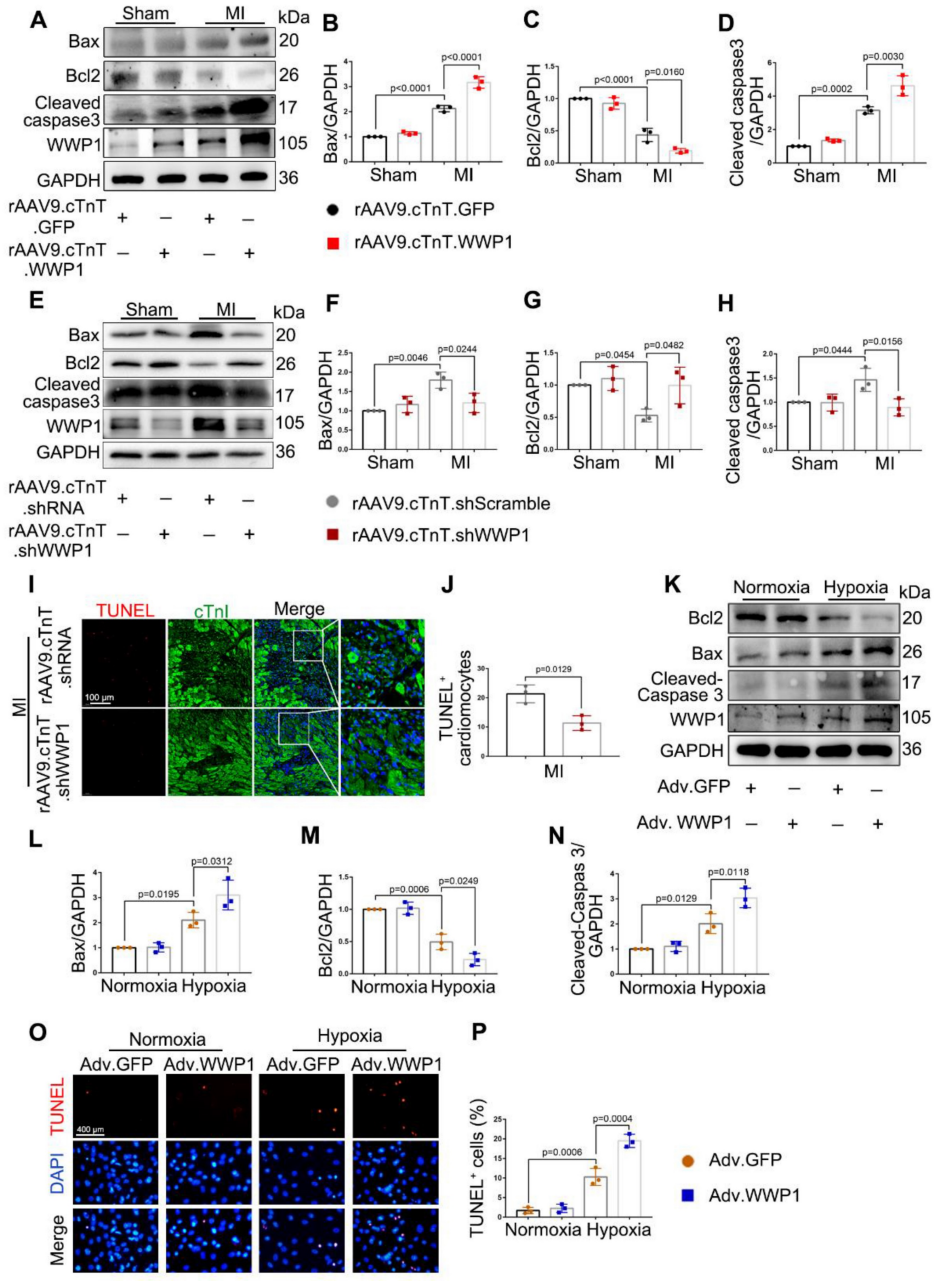
** WWP1 induces cardiomyocyte apoptosis post-MI. A-D WT mice were treated with rAAV9-cTnT-GFP or rAAV9-cTnT-WWP1 by intravenous injection of tail two weeks before suffering from sham or LAD ligation, and after additional one day, mice were sacrificed.** Representative Western blots and statistical results of Bax, Bcl2 and cleaved-caspase3 in the infarct zone. n = 3. **E-H** Mice were treated with rAAV9-cTnT-shScramble or rAAV9-cTnT-shWWP1 by intravenous injection of tail two weeks before suffering from sham or LAD ligation, and after additional three days, mice were sacrificed. Representative Western blots and statistical results of Bax, Bcl2 and cleaved-caspase3 in the infarct zone. n = 3. **I** TUNEL assay and immunofluorescence staining with cTnI were performed to detect cardiomyocyte apoptosis in the myocardium infected with rAAV9-cTnT-shScramble or rAAV9-cTnT-shWWP1. Scale bar = 100 μm. The enlarged image represents a digital enlargement of the area indicated within the box. n = 3. **J** Statistical results of TUNEL+ cardiomyocytes at day 3 post-MI. K-N NRCMs infected with Adv-GFP or Adv-WWP1 for 48 h followed by hypoxic stimulation for 6 h. Representative Western blots and statistical results of Bax, Bcl2 and cleaved-caspase3 in NRCMs. n = 3. **O,** P TUNEL assay and statistical result of TUNEL^+^ cardiomyocytes. Scale bar = 400 μm. n = 3. The data are shown as the means ± SD. The data shown in B-D, F-H, J, L-N, and P were analysed by one-way ANOVA followed by Bonferroni post hoc test.

Apoptosis is known to be the early form of active and controllable cardiomyocyte death post-MI 27. The role of WWP1 in controlling apoptosis of cardiomyocytes, and apoptotic phenotypes were evaluated at day 1 and day 3 post-MI. Cardiomyocyte-specific overexpressed WWP1 led to elevated levels of pro-apoptotic proteins as Bax and cleaved-caspase 3, but a decrease in anti-apoptotic protein Bcl2 was observed at day 1 post-MI (Figure 3A-D). In contrast, cardiomyocyte-specific inhibition of WWP1 dramatically reversed the expression of apoptosis-related proteins at day 3 post-MI (Figure 3E-H). TUNEL assay and immunofluorescence staining detected apoptosis of cardiomyocytes manifested as TUNEL-positive apoptotic nuclei co-stained with cTnI. Consistant with the expression changes of apoptosis-related proteins, WWP1 knockdown significantly reduced TUNEL-positive cardiomyocytes at day 3 post-MI; however, apoptotic cells were not observed in sham-mice (Figure 3I-J, Figure S2).

The effect of up-regulated WWP1 on cardiomyocyte apoptosis was further investigated by performing *in vitro* experiments. NRCMs were infected with Adv-WWP1 or Adv-GFP for 48 h before undergoing hypoxia stimulation for 6 h. Western bolt analysis and the TUNEL assay revealed an increased level of apoptosis in hypoxia-treated NRCMs provoked by WWP1 overexpression (Figure 3K-P). Like the* in vivo* observations, *in vitro* data also supported that high expression level of WWP1 in hypoxia-treated cardiomyocytes contributes to exacerbated cardiomyocyte apoptosis.

### WWP1 governs cardiomyocyte inflammatory response post-MI

**Figure 4:**
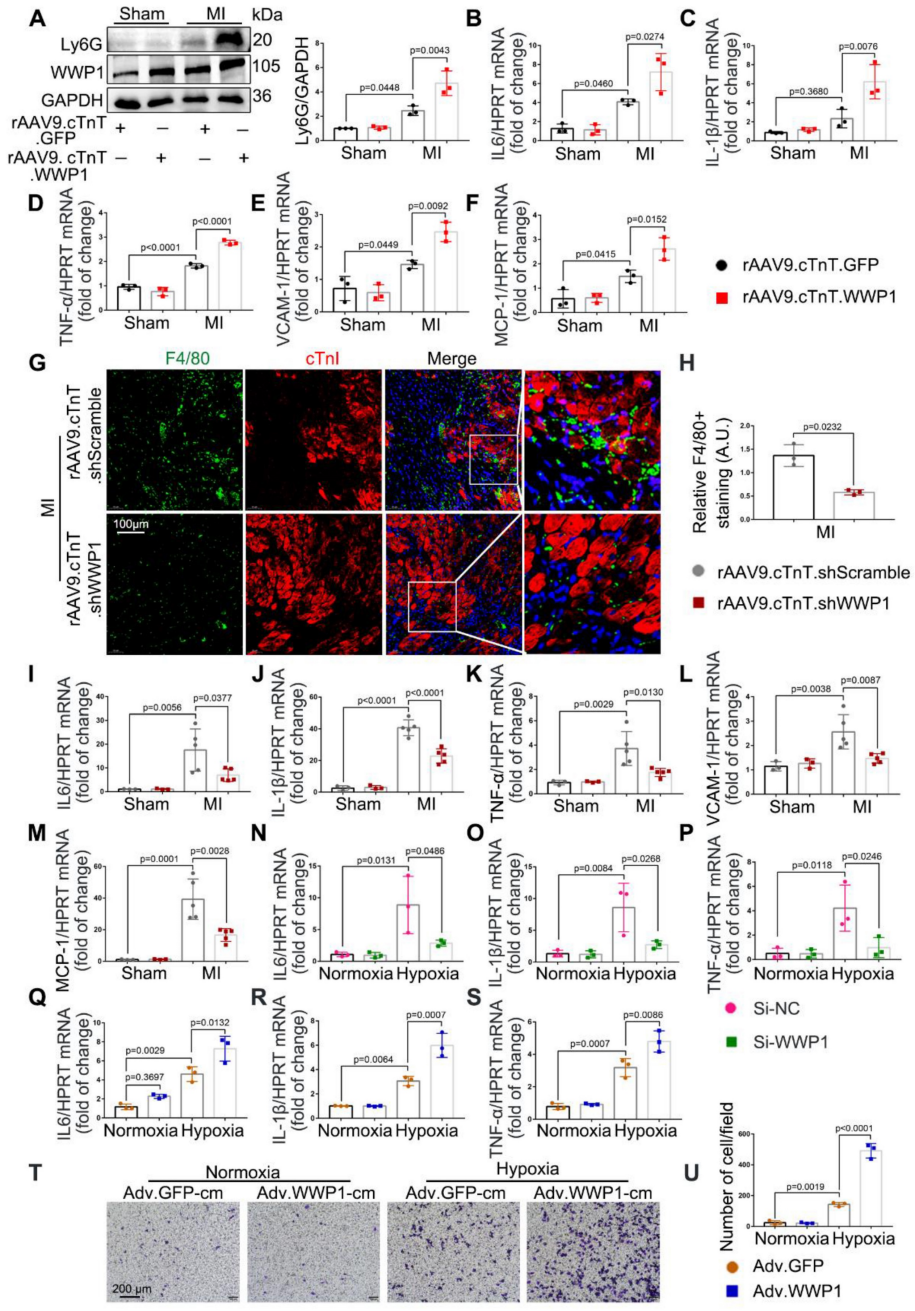
** WWP1 triggers cardiomyocyte inflammatory response post-MI. A** WT mice were treated with rAAV9-cTnT-GFP or rAAV9-cTnT-WWP1 by intravenous injection of tail two weeks before suffering from sham or LAD ligation, and after additional one day, mice were sacrificed. Representative Western blot and statistical result of Ly6G in the infarct zone. n = 3. **B-F** mRNA levels of IL-6, IL-1β, TNF-α, VCAM-1, and MCP-1 in infarcted hearts or healthy hearts were tested by qRT-PCR. n = 3.** G, H** Mice were treated with rAAV9-cTnT-shScramble or rAAV9-cTnT-shWWP1 by intravenous injection of tail two weeks before suffered from sham or LAD ligation, and after additional three day, mice were sacrificed. Immunofluorescence co-staining for cTnI with F4/80 and DAPI in infarcted hearts. Scale bar = 100 μm. The enlarged image represents a digital enlargement of the area indicated within the box. n = 3. **I-M** mRNA levels of IL-6, IL-1β, TNF-α, VCAM-1, and MCP-1 in infarcted or healthy hearts were tested by qRT-PCR. n = 3, 3, 5, 5, respectively. **N-P** H9C2 cells were transfected with Si-NC or Si-WWP1 for 48 h followed by hypoxic stimulation for 6 h. mRNA levels of IL6, IL-1β, and TNF-α were tested by qRT-PCR. n = 3. **Q-S** NRCMs infected with Adv-GFP or Adv-WWP1 for 48 h followed by hypoxic stimulation for 6 h. mRNA levels of IL6, IL-1β, and TNF-α were tested by qRT-PCR. n = 3. **T** NRCMs infected with Adv-GFP or Adv-WWP1 for 48 h followed by hypoxic stimulation for 6 h. Conditioned media was collected and used to treat RAW264 cells. Transwell assay was performed to test the chemotaxis of RAW264 cells. Scale bar = 200 μm. n = 3. The data are shown as the means ± SD. The data shown in A-U were analysed by one-way ANOVA followed by Bonferroni post hoc test.

Accumulative evidence from previous studies showed that excessively activated inflammatory response is a pivotal mechanism leading to cardiac ischemic injury at the incipient stage of MI 28-30. Following myocardial infarction, days 1-3 are generally considered the early inflammatory phase during which locally secreted chemokines and inflammatory factors recruit leucocytes into the damaged myocardium 16, with neutrophil infiltration mainly on the day 1 followed by macrophage infiltration as the predominant immune cells at day 3 26. We observed that cardiomyocyte-specific overexpressed WWP1 significantly increased Ly6G abundance and slightly increased CD68 expression in the infarcted myocardium, attributed to higher mRNA levels of IL-6, IL-1β, TNF-α, VCAM-1, and MCP-1 (Figure 4A-F, Figure S3A). Immunofluorescence co-staining with cTnI in the infarcted heart tissues demonstrated that F4/80^+^ macrophages were mostly recruited near surviving cardiomyocytes in the infarct/border zone, as reported previously 26. Conversely, cardiomyocyte-specific WWP1 knockdown inhibited infarcted heart tissues from harboring excessive F4/80^+^ macrophages at day 3 post-MI (Figure 4G-H, Figure S3C), along with a moderately mitigated Ly6G protein level (Figure S3B), remarkably reducing mRNA levels of inflammatory mediators (Figure 4I-M). We performed *in vitro* experiments using Si-RNA or Adv-WWP1. qRT-PCR revealed that knockdown of WWP1 in H9C2 cells reduced expression of multiple pro-inflammatory cytokines, including IL-1β, IL-6, and TNF-α after hypoxia treatment for 6 h, but overexpressing WWP1 led to the opposite effect (Figure S4A, Figure 4N-S). Subsequently, we assessed whether the secretion of pro-inflammatory cytokines from cardiomyocytes induced by WWP1 could affect the chemotaxis of macrophages. Conditioned media collected from hypoxia-treated NRCMs were used to treat RAW264 cells. Transwell assay showed that the conditioned medium from hypoxia-treated NRCMs expressing GFP (Adv-GFP-cm) induced apparent chemotaxis of RAW264 cells, and the effect was potentiated when WWP1 was overexpressed (Adv-WWP1-cm) (Figure 4T-U). Taken together, these data substantiated the crucial role of WWP1 in manipulating the inflammatory response of cardiomyocytes.

### WWP1 degrades KLF15 by catalyzing K48-linked polyubiquitination in hypoxia-treated cardiomyocytes

**Figure 5:**
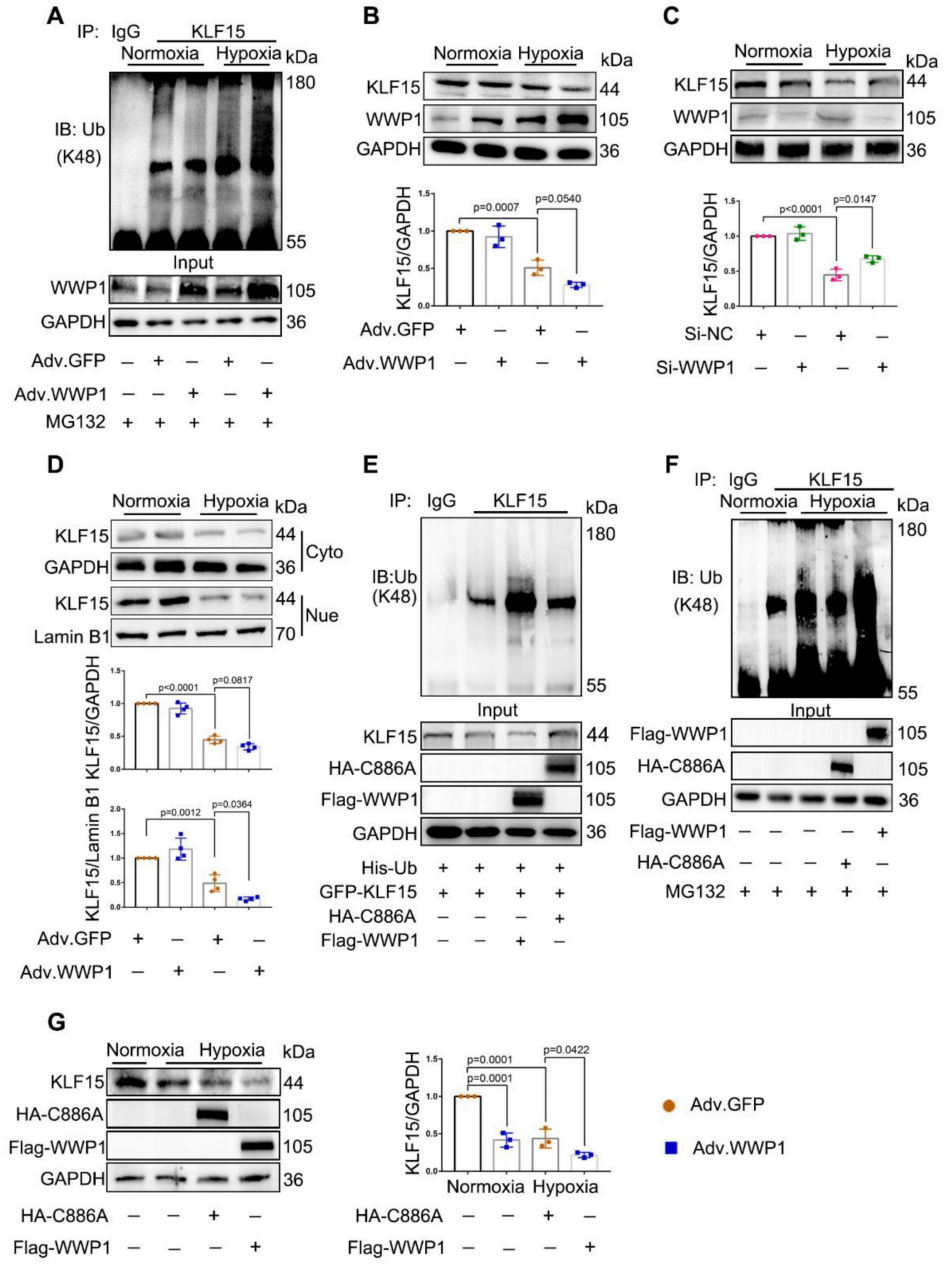
** WWP1 promotes KLF15-ubiquitination mediated degradation in hypoxia-induced cardiomyocytes. A** NRCMs were infected with Adv-WWP1 or Adv-GFP for 48 h before the cells were treated with hypoxia and MG132 (5 μM) for 6 h, synchronously. Cellular proteins were isolated for immunoprecipitation with anti-KLF15 antibody followed by immunoblot with anti-Ub-K48 antibody. IgG as a negative control. n = 3.** B** Total expression level of KLF15 in NRCMs were examined by Western blots. Corresponding statistics of KLF15 were shown. n = 3. **C** H9C2 cells were transfected with Si-NC or Si-WWP1 for 48 h followed by hypoxic stimulation for 6 h. Total expression level of KLF15 were examined by Western blots. Corresponding statistics of KLF15 were shown. n = 3.** D** Expression levels of KLF15 in cytoplasm and nucleus in NRCMs were examined by Western blots. Corresponding statistics of KLF15 in cytoplasm and nucleus were shown. n = 3. **E** HEK293T cells transfected with vectors for His-Ub, GFP-KLF15, and either Flag-WWP1 or HA-C8886A mutant form of WWP1 were subjected to immunoprecipitation with the antibody against KLF15, and followed by immunoblot with anti-Ub-K48 antibody. IgG as a negative control. n = 3. **F** H9C2 cells were transfected with Flag-WWP1 plasmid or HA-C886A plasmid for 36 h before synchronous administration with hypoxia and MG132 (5 μM) for 6 h. Immunoprecipitation with the antibody against KLF15, and followed by immunoblot with anti-Ub-K48 antibody. IgG as a negative control. n = 3. **G** H9C2 cells were transfected with Flag-WWP1 plasmid or HA-C886A plasmid for 36 h before administration with hypoxia for 6 h. Total expression level of KLF15 were examined by Western blots. Corresponding statistics of KLF15 were shown. n = 3. The data are shown as the means ± SD. The data shown in B-D, and G were analysed by one-way ANOVA followed by Bonferroni post hoc test.

WWP1 has recently been identified as a negative regulator of KLF15, suppressing its activation via ubiquitination and degradation 25. We investigated whether KLF15 was involved in mechanisms underlying WWP1-mediated inflammatory response in damaged cardiomyocytes. We found that WWP1 overexpression strikingly attenuated KLF15 protein level in hypoxia-treated NRCMs, along with enhanced ubiquitination at lysine-48 (K48) residue of KLF15 (Figure 5A-B). On the contrary, WWP1 knockdown up-regulated KLF15 protein level in hypoxia-treated H9C2 cells (Figure 5C). Reduced protein levels of KLF15 were evident in the nuclei from hypoxia treated NRCMs after WWP1 overexpression (Figure 5D, Figure S7). Also, increased WWP1 did not influence the ubiquitin modification and protein abundance of KLF15 in the normoxia-treated control groups.

Whether the hypoxia-induced diminished KLF15 due to the catalytic activation of WWP1 was determined by using a WWP1 mutant in which cysteine-886 (C886A) was replaced by alanine to abolish E3 ubiquitin ligase activity. HEK293T cells transduced with His-Ub, GFP-KLF15, and either Flag-WWP1 or HA-WWP1 (C8886A) mutant vectors were immunoprecipitated with the antibody against KLF15. Co-transfection of His-Ub and Flag-WWP1 increased polyubiquitination and reduced KLF15, whereas no detectable alterations in the KLF15 polyubiquitination level and total amount were discovered after overexpression of catalytically inactive WWP1 mutant (C886A) (Figure 5E). Next, H9C2 cells were transduced with Flag-WWP1 or HA-C886A plasmid before hypoxia treatment. Increased WWP1 expression further promoted ubiquitin modification and degradation of KLF15 in the hypoxic condition, whereas no difference in ubiquitination was observed between the control and C886A groups after hypoxia (Figure 5F-G). These results suggested that the E3 ubiquitin ligase activity of WWP1 targets KLF15 in cardiomyocytes, resulting in its K48-linked polyubiquitination and degradation.

### WWP1 triggers inflammatory activation signals of NF‑κB and MAPK in hypoxia-treated cardiomyocytes

**Figure 6:**
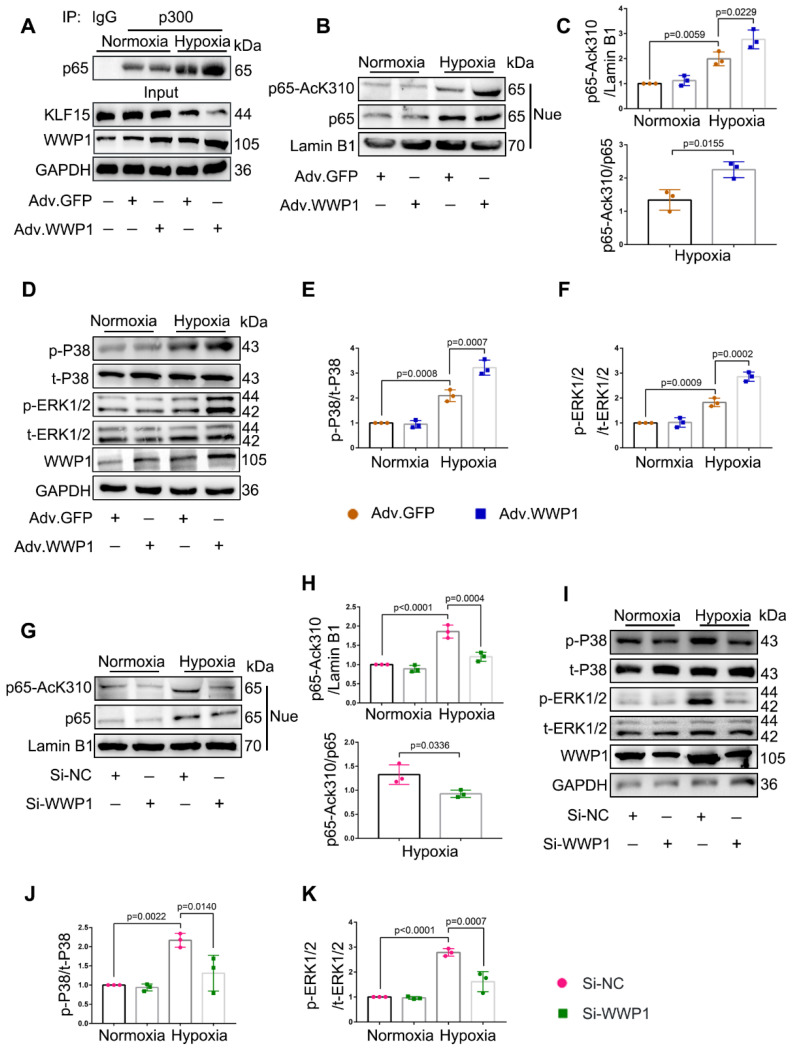
** WWP1 promotes inflammatory activation signals of NF‑κB and MAPK in hypoxia-induced cardiomyocytes. A** Interaction of p300 with p65 was determined by immunoprecipitation with anti-p300 antibody followed by immunoblot with anti-p65 antibody. IgG as a negative control. n = 3. **B, C** Western blot analysis and statistical results of expression of P65-AcK310 in nucleus. n = 3. **D-F** The levels of phosphorylated P38 and ERK1/2 were examined by Western blots, and statistical results were shown. n = 3. **G, H** H9C2 cells were transfected with Si-NC or Si-WWP1 for 48 h followed by hypoxic stimulation for 6 h. Western blot analysis and statistical results of expression of P65-AcK310 in nucleus. n = 3. **I-K** The levels of phosphorylated P38 and ERK1/2 in H9C2 cells under the condition of WWP1 knockdown were examined by Western blots, and statistical results were shown. n = 3. The data are shown as the means ± SD. The data shown in C, E, F, H, J, and K were analysed by one-way ANOVA followed by Bonferroni post hoc test.

Following WWP1 overexpression, increased pro-inflammatory factors in cardiomyocytes, including IL-6, IL-1β, and TNF-α, are established targets of NF-κB transcriptional signaling pathway involved in the inflammatory response 31, suggesting a possible involvement of NF-κB activation in WWP1-induced cardiomyocyte inflammation after MI. Meanwhile, KLF15 was reported to inhibit NF-κB transcriptional activation by suppressing p300-mediated acetylation 32. Thus, we performed a co-immunoprecipitation analysis to detect the interaction between p300 and p65 in NRCMs exposed to hypoxia, revealing an increased association of p300 with p65. However, overexpression of WWP1 further enhanced the interaction between p300 and p65 (Figure 6A).

Next, we sought to explore the significance of KLF15-dependent NF-κB acetylation. As expected, compared to NRCMs treated with the control virus, overexpression of WWP1 in cardiomyocytes caused increased p65 acetylation (AcK310-p65) (Figure 6B-C), but did not affect total p65 in the nucleus in a hypoxic environment (statistical chart not shown). It was reported that KLF15 inhibits the MAPK signaling pathway in cardiomyocytes 24. Therefore, we examined the effect of increased expression of WWP1 on MAPK signaling activation. Hypoxia treatment markedly increased the phosphorylation level of P38 and ERK1/2 compared with the control, and increased WWP1 expression further activated the MAPK signaling pathway in hypoxia-treated NRCMs (Figure 6D-F). Conversely, WWP1 knockdown weakened the activation signal of NF‑κB and MAPK induced by hypoxia (Figure 6G-K). These data demonstrated that WWP1 is a critical regulator for down-regulating of KLF15 and subsequently activates inflammatory signals of NF‑κB and MAPK in hypoxia-treated cardiomyocytes.

### Inhibition of WWP1 expression in cardiomyocytes restrains KLF15-mediated inflammatory signals in the infarcted myocardium

**Figure 7:**
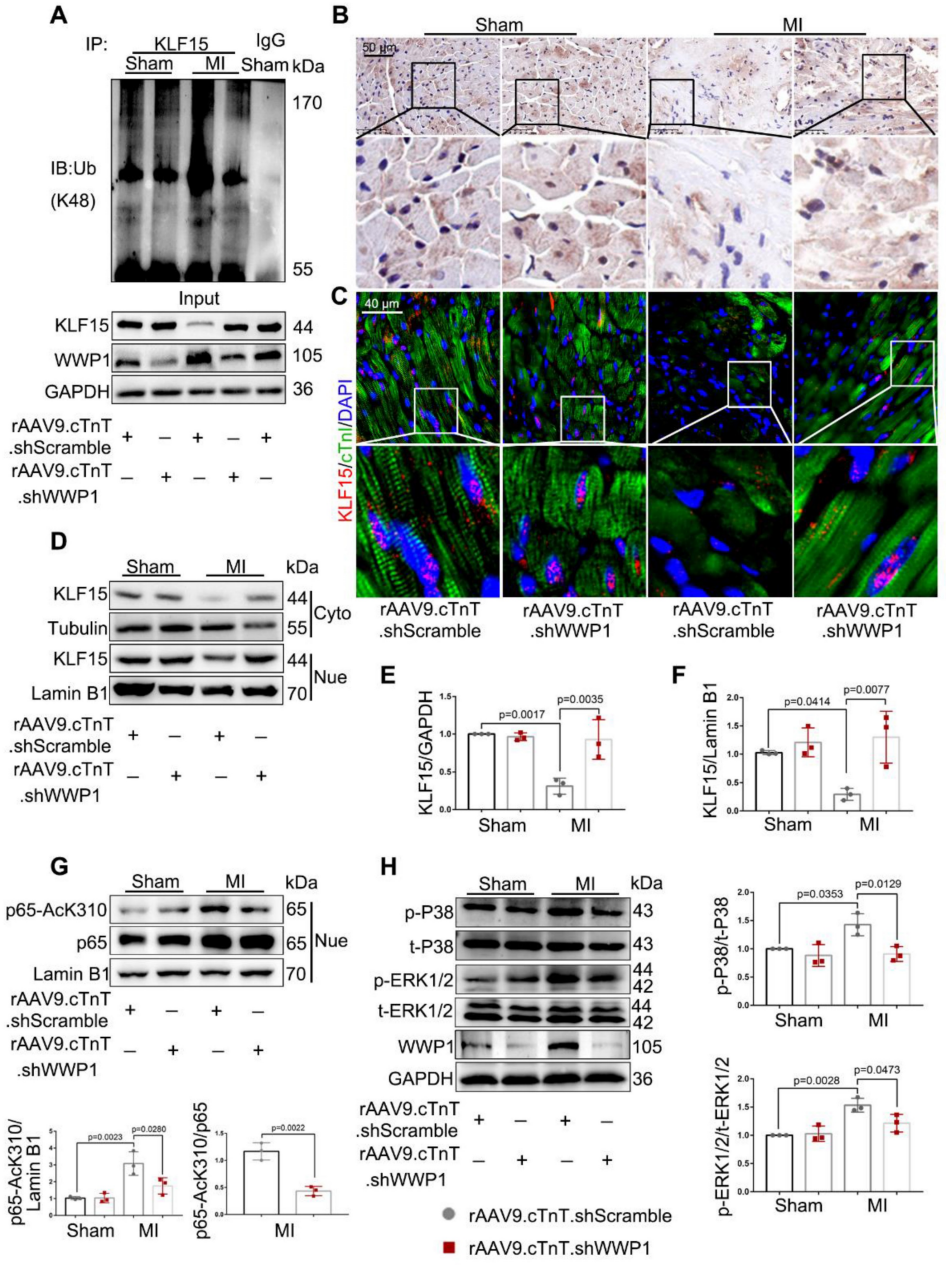
**Inhibition of WWP1 expression in cardiomyocytes restrains NF-κB and MAPK activation involving degrading KLF15 in the infarcted myocardium.** Mice were treated with rAAV9-cTnT-shScramble or rAAV9-cTnT-shWWP1 by intravenous injection of tail two weeks before suffered from sham or MI surgery, and after additional one day, mice were sacrificed. **A** Ubiquitination of KLF15 was detected by immunoprecipitation with anti-KLF15 antibody followed by immunoblot with anti-Ub-K48 antibody. IgG as a negative control. n = 3. **B** Immunohistochemistry for KLF15 (brown) in sham or infarcted hearts. Scale bar = 50 μm. n = 3. **C** Immunofluorescence co-staining for cTnI with KLF15 and DAPI in mice hearts post-MI. Scale bar = 40 μm. n = 3. **D-F** Protein expression of KLF15 in cytoplasm and nuclear lysates harvested from infarct areas post-MI was detected by Western blots. Corresponding statistics of KLF15 were shown. n = 3. **G** Western blot analysis and statistical results of expression of P65-AcK310 in nucleus. n = 3. H The levels of phosphorylated P38 and ERK1/2 were examined by Western blots, and statistical results were shown. n = 3. The data are shown as the means ± SD. The data shown in E-H were analysed by one-way ANOVA followed by Bonferroni post hoc test.

Consistent with the *in vitro* results, a dramatically elevated ubiquitination level of KLF15 was detected in the infarct area at day 1 post-MI. However, down-regulation of WWP1 markedly reduced KLF15 degradation through K48-linked ubiquitination (Figure 7A). Immunohistochemistry and immunofluorescence analysis showed that KLF15 was present in the cytoplasm and located in the nucleus predominantly in cardiomyocytes from sham-operated mice but was significantly decreased in the infarct area (Figure 7B-C). Inhibition of WWP1 expression remarkably increased KLF15 in cardiomyocytes located in the peri-infarct zone (Figure 7C). Western blotting detected elevated KLF15 protein levels in the cytoplasm and nucleus in MI-mice with cardiomyocyte-specific WWP1 knockdown (Figure 7D-F, Figure S7). Down-regulation of WWP1 markedly prevented MI-induced acetylation of p65 and phosphorylation of P38 and ERK1/2 (Figure 7G-H). These findings suggested that WWP1 in cardiomyocytes is critical in promoting KLF15 ubiquitination and degradation, resulting in enhanced NF-κB and MAPK activation in the infarcted myocardium.

### KLF15 is essential for the regulatory function of WWP1 in cardiac ischemic injury post-MI

**Figure 8:**
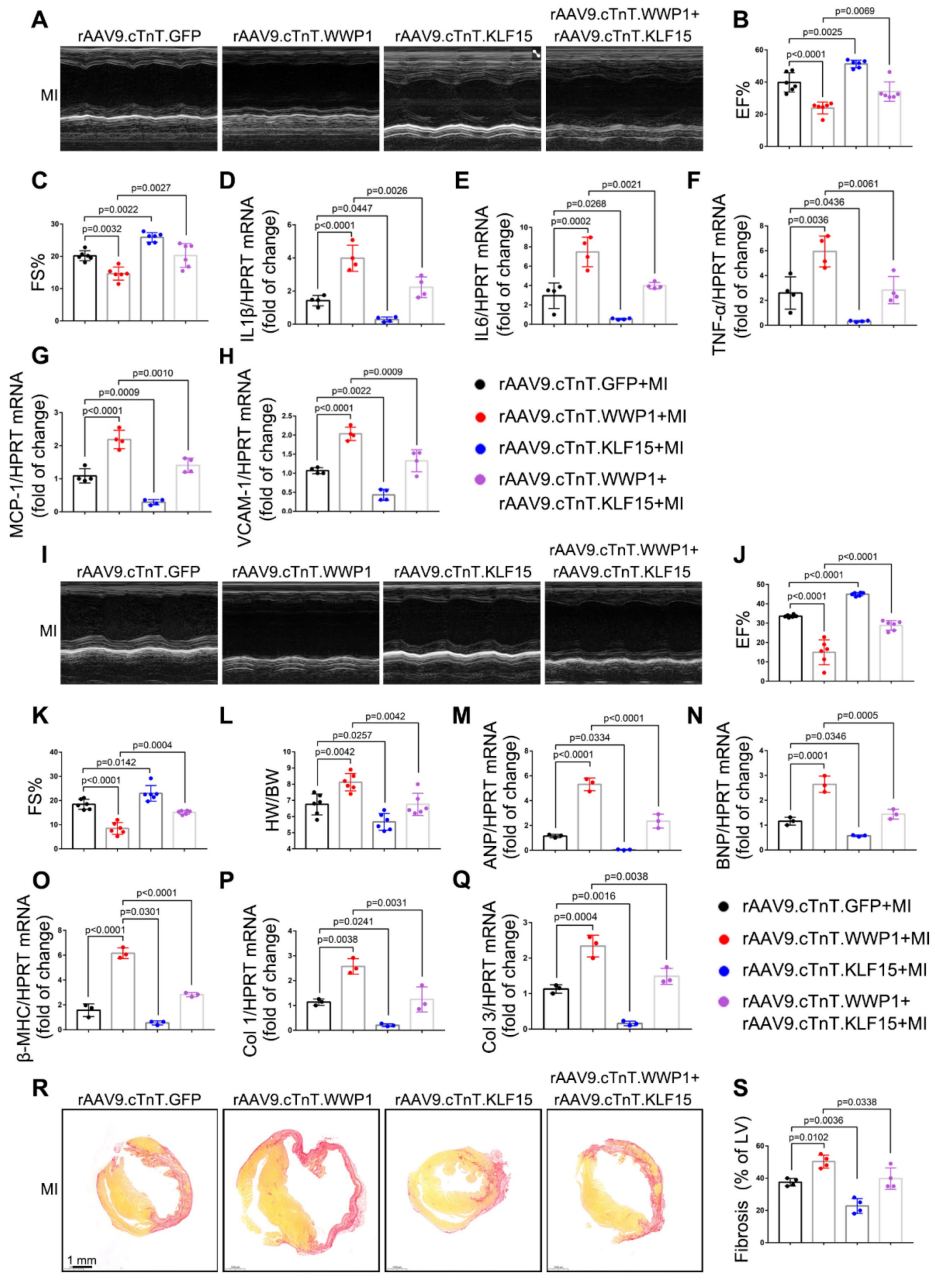
**The regulatory function of WWP1 on cardiac ischemic injury post-MI is dependent on KLF15.** Mice were injected with rAAV9-cTnT-WWP1 with or without rAAV9-cTnT-KLF15 before they subjected to MI. **A-C** Cardiac function indices were measured by echocardiography at day 3 post-MI. n = 6. **D-H** mRNA levels of IL-6, IL-1β, TNF-α, VCAM-1, and MCP-1 in infarcted or healthy hearts were tested by qRT-PCR at day 3 post-MI. n = 4. **I-K** Cardiac function indices were measured by echocardiography at day 21 post-MI. n = 6. **L** Ratio of heart weight/ body weight (HW/BW). n = 6. **M-Q** qRT-PCR was used to test the mRNA levels of ANP, BNP, β-MHC, collagen I, collagen III. n = 3. **R, S** Sirius red staining were performed to detect fibrosis. n = 4. The data are shown as the means ± SD. The data shown in B-H, J-Q, and S were analysed by one-way ANOVA followed by Bonferroni post hoc test.

We engineered rAAV9 viral vectors under the control of the cardiomyocyte-specific cTnT promoter to overexpress KLF15 or knock down KLF15 in cardiomyocytes to verify the role of the KLF15 in MI regulation by WWP1. Mice were injected with rAAV9-cTnT-WWP1 with or without rAAV9-cTnT-KLF15 before subjecting them to MI for an additional 3 days. As predicted, cardiomyocyte-specific WWP1 overexpression in mice after MI resulted in marked cardiac dysfunction and inflammation. However, cardiomyocyte-specific overexpression of KLF15 reversed the influence of WWP1 on cardiac dysfunction and inflammation, as demonstrated by increased EF%, FS%, and lower levels of IL-6, IL-1β, TNF-α, VCAM-1, and MCP-1 at day 3 post-MI (Figure S4B, Figure 8A-H). In contrast, double inhibition of WWP1 and KLF15 expressions did not significantly improve ischemia-induced cardiac dysfunction (Figure S4C, Figure S5A-C). Cardiomyocyte-specific WWP1 overexpression also aggravated MI-induced cardiac dysfunction and ventricular remodeling at day 21 post-MI, but these protective effects were abolished when simultaneously overexpressing KLF15 (Figure 8I-S). These data suggested that KLF15 is essential for the regulatory function of WWP1 on cardiac ischemic injury post-MI.

### Targeting WWP1 suppresses myocardial inflammation post-MI

**Figure 9:**
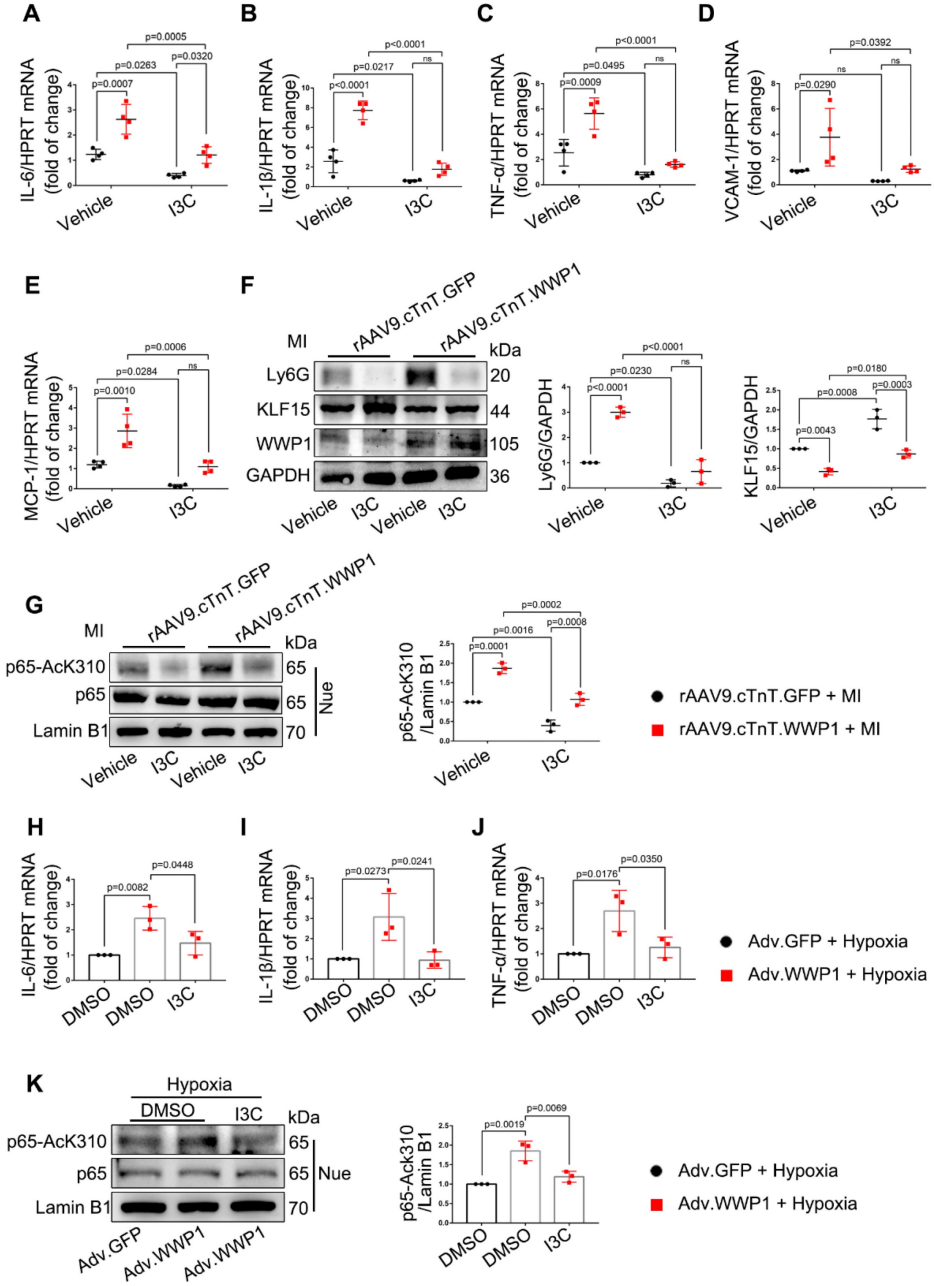
** I3C pre-treatment suppresses WWP1-mediated excessive myocardial inflammation post-MI.** Mice were administered with vehicle or I3C (20 mg/kg) three times a week for a month. When mice were pre-treated by I3C for two weeks, they were randomly assigned to rAAV9-cTnT-WWP1 or rAAV9-cTnT-GFP injection, and all of which were subjected to LAD ligation after two weeks of virus injection. All mice sacrificed 1day after induction of MI.** A-E** mRNA levels of IL-6, IL-1β, TNF-α, VCAM-1, and MCP-1 in infarcted or healthy hearts were tested by qRT-PCR. n = 4. **F** Representative Western blot and statistical result of Ly6G and KLF15 in the infarct zone. n = 3. **G** Western blot analysis and statistical results of expression of P65-AcK310 in nucleus. n = 3. **H-J** H9C2 cells were infected with Adv-WWP1 or Adv-GFP for 24 h followed by I3C (50 μM) treatment for 24 h, and then the cells were treated with hypoxia for 6 h. mRNA levels of IL-6, IL-1β, TNF-α were tested by qRT-PCR. n = 3. **K** Western blot analysis and statistical results of expression of P65-AcK310 in nucleus in H9C2 cells. n = 3. The data are shown as the means ± SD. The data shown in A-G were analysed by two-way ANOVA followed by Bonferroni post hoc test. **H-K** were analysed by one-way ANOVA followed by Bonferroni post hoc test.

Indole-3-carbinol (I3C), which can suppress E3 enzymatic activity of WWP1 33, was employed as a natural WWP1 inhibitor. We hypothesized that targeting WWP1 by I3C might play a potential functional role in preventing MI-induced cardiac dysfunction and remodeling by inhibiting myocardial inflammation. Mice were administered with vehicle or I3C (20mg/kg), randomly assigned to receive rAAV9-cTnT-WWP1 or control virus injection, and subsequently subjected to LAD ligation. I3C treatment reduced the WWP1 overexpression-mediated myocardial inflammatory response post-MI, as evidenced by decreased mRNA levels of IL-6, IL-1β, TNF-α, VCAM-1, and MCP-1, and reduced Ly6G protein level, accompanied by the increased protein level of KLF15 (Figure 9A-F). As expected, I3C treatment also suppressed WWP1-mediated up-regulation of AcK310-p65 (Figure 9G). Immunoblotting and ubiquitination assays were performed to examine that I3C (50 μM) inhibited WWP1-mediated KLF15 K48-linked polyubiquitination in hypoxia-treated H9C2 cells (Figure S6A-C). A shown in Figure 9H-J, I3C-mediated inhibition of WWP1 also prevented the production of inflammatory factors (Figure 9H-J) and suppressed inflammatory signals of NF‑κB and MAPK in hypoxia-treated H9C2 cells (Figure 9K, Figure S6D).

### Targeting WWP1 protects against cardiac dysfunction and remodeling after MI

**Figure 10:**
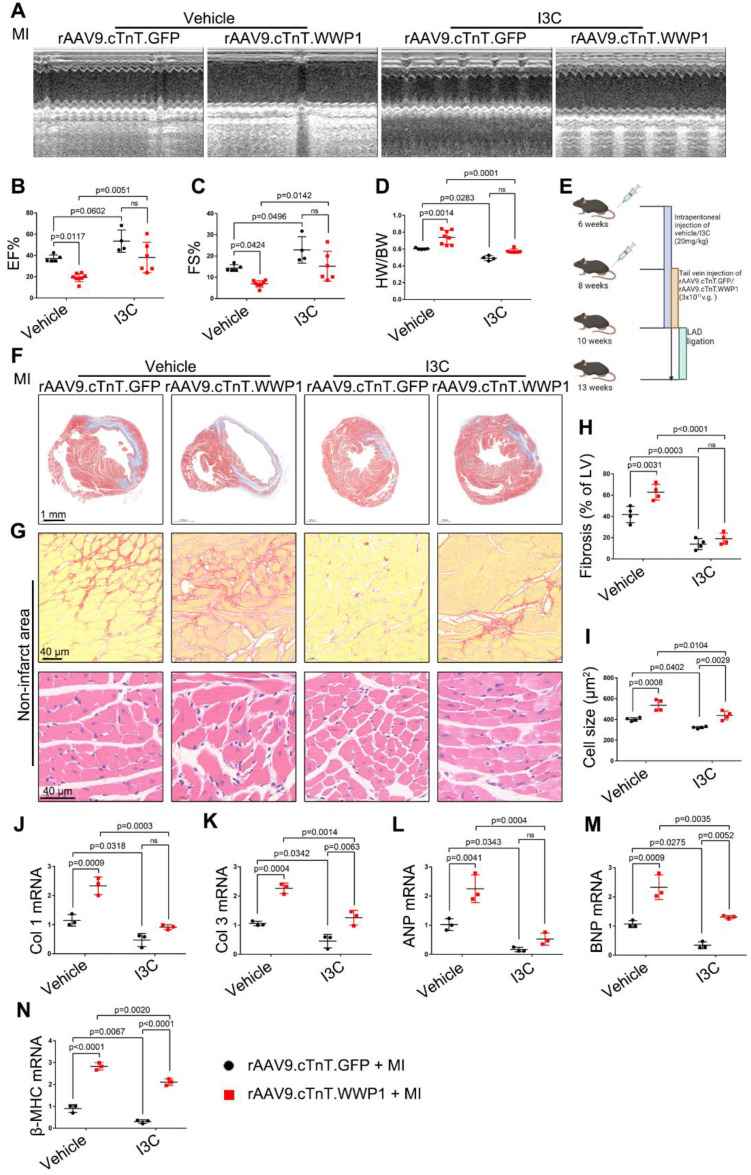
** I3C pre-treatment protects against WWP1-mediated cardiac dysfunction and remodeling after MI. A-C** Mice were administered with vehicle or I3C (20 mg/kg) three times a week for a month. When mice were pre-treated by I3C for two weeks, they were randomly assigned to rAAV9-cTnT-WWP1 or rAAV9-cTnT-GFP injection, and all of which were subjected to LAD ligation after two weeks of virus injection. All mice sacrificed two weeks after induction of MI. cardiac function indices were measured by echocardiography. EF% ejection fraction, FS% fractional shortening. n = 5, 4, 8, 6, respectively. **D** Ratio of heart weight/ body weight (HW/BW). n = 5, 4, 8, 6, respectively. **E** Treatment regimen. **F** Masson's trichrome staining was performed to detect the fibrosis in left ventricle. n = 4. **G** Sirius red staining and hematoxylin (top) and eosin (H&E) staining (bottom) were performed to detect fibrosis and hypertrophy in non-infarct zone. n = 4. **H, I** Corresponding fibrosis area and cardiomyocyte cross-sectional area were calculated. n = 4. **J-N** qRT-PCR was used to test the mRNA levels of collagen I, collagen III, ANP, BNP, β-MHC. n = 3. The data are shown as the means ± SD. The data shown in B-D, H-N were analysed by two-way ANOVA followed by Bonferroni post hoc test.

Next, we evaluated the long-term effect of targeting WWP1 on cardiac dysfunction and remodeling after MI (Figure 10E). Echocardiographic parameters revealed that I3C administration improved MI-induced cardiac dysfunction. Furthermore, I3C administration alleviated cardiomyocyte-specific WWP1 overexpression-induced deterioration of cardiac function post-MI (Figure 10A-C). The heart weight (HW)/body weight (BW) ratio in rAAV9.cTnT.GFP- or rAAV9.cTnT. WWP1-injected MI-mice was also reduced after I3C administration (Figure 10D). Morphological analysis demonstrated that I3C administration contributed to a less fibrotic area in infarcted myocardium in rAAV9.cTnT.GFP-and rAAV9.cTnT. WWP1-injected MI-mice (Figure 10F). Additionally, WWP1-induced interstitial fibrosis and cardiomyocyte cross-sectional area in the non-infarct areas were alleviated by I3C administration (Figure 10G-I). Excessive interstitial fibrosis and cardiomyocyte hypertrophy, evidenced by increased collagen I and III, atrial natriuretic peptide (ANP), brain natriuretic peptide (BNP), and β-myosin heavy chain (β-MHC) mRNA expression, were also suppressed by I3C administration (Figure 10J-N). These data suggested that WWP1 inhibitor I3C protects against cardiac dysfunction and remodeling after MI.

**Figure 11:**
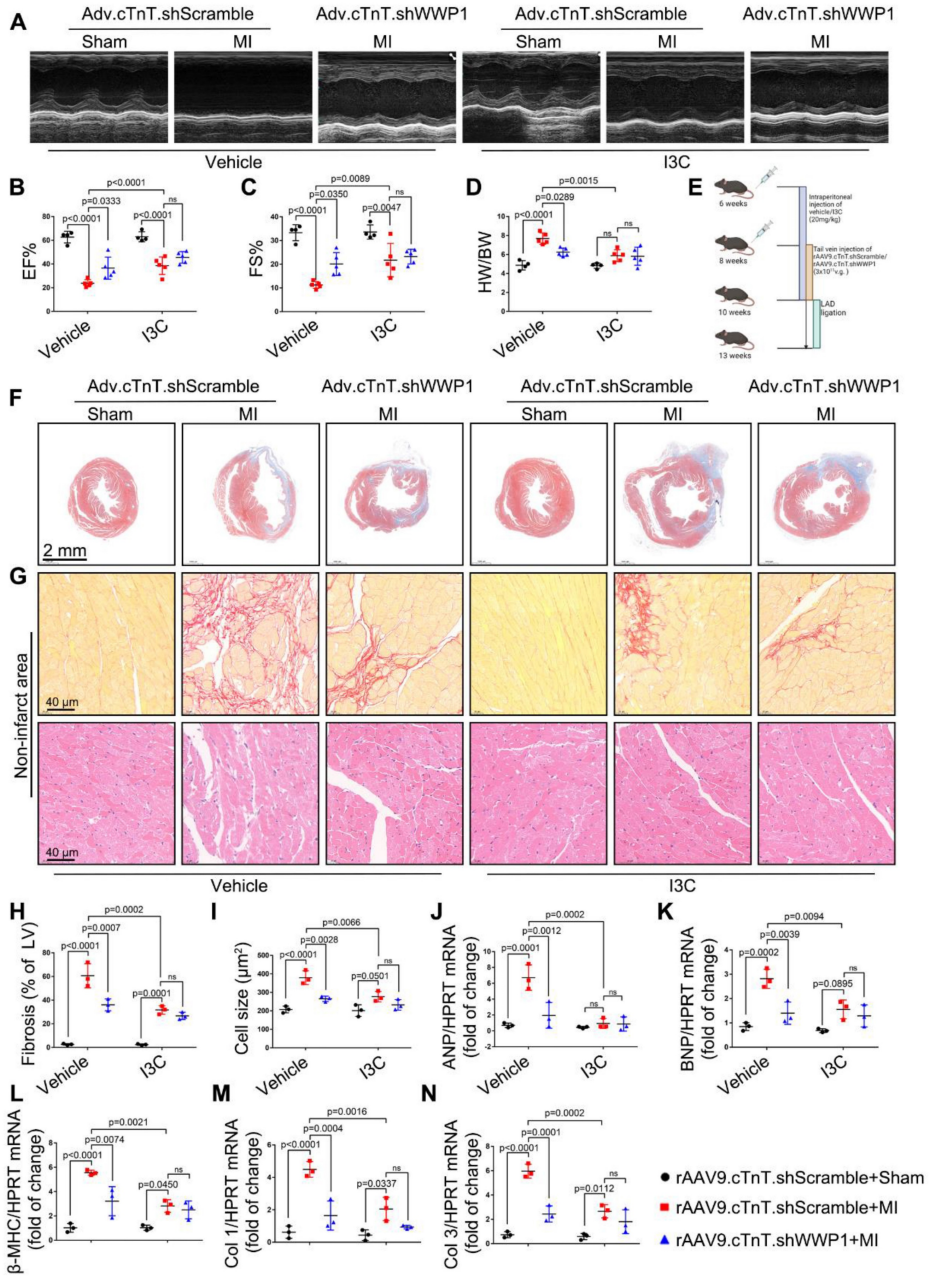
** I3C pre-treatment did not further improved cardiac function and remodeling after MI in WWP1-knockdown mice. A-C** Mice were administered with vehicle or I3C (20 mg/kg) three times a week for a month. When mice were pretreated by I3C for two weeks, they were randomly assigned to rAAV9-cTnT-shWWP1 or rAAV9-cTnT-shScramble injection, and all of which were subjected to LAD ligation after two weeks of virus injection. All mice sacrificed at day 21 after induction of MI. cardiac function indices were measured by echocardiography. EF% ejection fraction, FS% fractional shortening. n = 4, 5, 5, 4, 5, 5, respectively. **D** Ratio of heart weight/ body weight (HW/BW). n = 4, 5, 5, 4, 5, 5, respectively. **E** Treatment regimen. **F** Masson's trichrome staining was performed to detect the fibrosis in left ventricle. n = 3. **G** Sirius red staining and hematoxylin (top) and eosin (H&E) staining (bottom) were performed to detect fibrosis and hypertrophy in non-infarct zone. n = 3. **H, I** Corresponding fibrosis area and cardiomyocyte cross-sectional area were calculated. n = 3. **J-N** qRT-PCR was used to test the mRNA levels of collagen I, collagen III, ANP, BNP, β-MHC. n = 3. The data are shown as the means ± SD. The data shown in B-D, H-N were analysed by two-way ANOVA followed by Bonferroni post hoc test.

We examined the effect role of I3C in WWP1 knockdown condition mice to better understand its inhibitory actions (Figure 11E). MI markedly decreased EF% and FS% and increased the ratio of HW/BW in mice treated with control virus, but in rAAV9.cTnT.shWWP1-treated mice, opposite effects were observed at day 21 post-MI. Importantly, I3C administration alone and I3C in WWP1 knockdown mice displayed a similar cardiac function, and the ratio of HW/BW (Figure 11A-D). Similarly, the morphological analysis demonstrated that WWP1 knockdown contributed to a less fibrotic area in infarcted myocardium, and interstitial fibrosis and cardiomyocyte cross-sectional area in the non-infarct area, evidenced by down-regulated mRNA levels of collagen I and III, ANP, BNP, and β-MHC (Figure 11F-N). There were also no differences in these indicators in the I3C-MI-group and I3C-WWP1 knockdown-MI-group (Figure 11F-N). These results demonstrated that I3C targeted WWP1 to exert preventive therapeutic efficacy, but the combination of WWP1 knockdown and I3C treatment did not show significant additive effects compared to I3C administration or WWP1 knockdown alone.

## Discussion

The present study illustrated an unrecognized role of WWP1 in MI-induced myocardial injury. The significant finding of this study is that ischemia or hypoxia up-regulated WWP1 expression in cardiomyocytes, leading to deteriorated cardiac function and increased infarct size, while cardiomyocyte-specific inhibition of WWP1 alleviated myocardial injury. Our study is the first to suggest that increased expression of WWP1 is one of the key factors involved in post-MI cardiomyocyte inflammation. Mechanistically, WWP1 mediated ubiquitin-dependent degradation of KLF15 and promoted subsequent activation of NF-κB and MAPK, contributing to myocardial inflammation and loss of cardiomyocytes post-MI. An inhibitory of WWP1, I3C, could protect against cardiac dysfunction and remodeling after MI by targeting WWP1-mediated inflammatory progression. Thus, our study underscores the importance of WWP1 in governing the inflammatory response of cardiomyocytes post MI and identifies WWP1 as a promising therapeutic target for MI.

Although WWP1 is pivotal during the progression of cardiovascular diseases, its role in the heart is not fully understood. Recent studies have reported that WWP1 is involved in cardiac hypertrophy 20, atrial fibrosis 34, arrhythmogenesis 11 and heart failure (HF) with a preserved ejection fraction (EF; HFpEF) 12; however, these are non-ischemic cardiovascular diseases. Thus far, the role of WWP1 in ischemic cardiomyopathy remains unclear. Our data have uncovered a previously unappreciated role of WWP1 in regulating myocardial ischemic injury. This seminal study has verified that WWP1 is highly expressed in cardiomyocytes, and reported its role in regulating cardiomyocyte phenotypes may help identify novel targets for therapeutic intervention during the progression of heart diseases. The expression of WWP1 was reported to increase in response to pressure overload, microgravity, and age 10. Here, we observed that ischemia and hypoxia contributed to dramatic elevation of WWP1 in cardiomyocytes. Our work has delineated the pro-inflammatory functional role of WWP1 in regulating acute myocardial ischemic injury and identified KLF15 as a molecular target of WWP1. Our data supported WWP1 is detrimental to cardiomyocyte survival by manipulating the inflammatory response in the early stage of MI, which affects later ventricular remodeling.

Considerable advances have been made toward understanding the complex biological pathways manipulating the inflammation of cardiomyocytes post-MI 35. Necrotic cardiomyocytes in the infarct area provide a major stimulus for the post-infarction inflammatory response by releasing danger-associated molecular patterns (DAMPs) 36. DAMPs binding to pattern recognition receptors (PRRs) induce inflammatory pathways and stimulate cytokine secretion 37. Surviving cardiomyocytes in the peri-infarct zone adjoining the necrotic core are strategically positioned to signal to immune cells, vascular, and other interstitial cells that migrate to the region 38. Within hours, a regulated inflammatory response consisting mainly of neutrophils and monocyte/macrophages is initiated in the myocardium to remove dead cells and matrix debris 38. In addition, apoptotic cardiomyocytes induced by myocardial inflammatory response are present in the surviving portion of the wall adjacent to the ischemic area 39. The present study has shown that WWP1 governs an inflammatory program in cardiomyocytes to increase myocardial inflammation and apoptosis after MI. Our study also showed that hypoxia-induced inflammatory cytokine production in cardiomyocytes and increased the chemotaxis of macrophages after overexpression of WWP1* in vitro*. We found that WWP1 promoted apoptosis of cardiomyocytes, while inhibiting WWP1 reduced the number of apoptotic cardiomyocytes. To the best of our knowledge, this is the first study to identify cardiomyocyte WWP1 as a pro-inflammatory regulator of cardiomyocyte fate and myocardial injury.

UPS has been documented as a post-translational modification contributing to the pathogenesis and progression of multiple cardiovascular diseases 3. The importance of E3 ligases in myocardial ischemia injury has been well established. Here, we demonstrated that E3 ligase WWP1 targeting KLF15 in cardiomyocytes aggravated myocardial ischemia injury. Increasing evidence indicates that KLF15 plays a vital role in maintaining cardiac function 40, 41. Recent studies have revealed a vital role for KLF15 as an effective therapeutic target for the treatment of cardiovascular diseases due to its effects on transcriptional factors such as myocyte enhancer factor 2 (MEF2), GATA-binding protein 4 (GATA4), and myocardin 42. Additionally, KLF15 inhibits the expression of TGF-β, CTGF, and MRTF-A in cardiac fibroblasts to restrict the progression of cardiac fibrosis 42. Our data suggested that myocardial ischemia or hypoxia induced down-regulation of KLF15 expression in cardiomyocytes, led to cardiomyocyte inflammatory response. WWP1-mediated K48-linked ubiquitination of KLF15 reduced its expression in hypoxia-administrated cardiomyocytes. Although WWP1 has been identified as a KLF15 regulator in skeletal muscle and C2C12 myoblasts 25, the WWP1/KLF15 axis signaling has not been clarified. KLF15 alters the acetylation status and activity of the pro-inflammatory factor NF-κB through direct interaction with the histone acetyltransferase p300 22. In this study, we revealed that WWP1 did not significantly alter the expression of p65 in the nucleus, but augmented the acetylation level of p65 in cardiomyocytes treated with hypoxia, indicating degradation of KLF15 catalyzed by WWP1 resulted in increased NF-κB acetylation that may enhance NF-κB transcriptional activity. We also found that WWP1 enhanced the phosphorylation of P38 and ERK1/2 in hypoxia-treated cardiomyocytes. The activation of MAPK by dual phosphorylation was correlated with the inflammatory phenotype, and targeted intervention of this pathway improved the prognosis of MI by interfering with the occurrence and development of inflammation 43. These data indicated that WWP1 mediated KLF15/NF-κB and MAPK inflammatory program is one of the leading causes of inflammatory response and sudden massive loss of cardiomyocytes.

Patients surviving from myocardial infarction experience ventricular remodeling and heart failure. The loss of cardiomyocytes and intense inflammation response following the infarction-overwhelms the limited regenerative capacity of the myocardium 44. I3C, a natural compound existing in cruciferous vegetables with negligible toxicity, was reported to target and inactivate WWP1^33^, and has been demonstrated to concentrate in the heart, lung, liver, kidney and brain 45. I3C was rapidly absorbed and eliminated from the plasma and tissues within one hour after administering an oral dose of 200 mg/kg in CD-1 mice 45. Also, at an intraperitoneal dose of 20 mg/kg three times a week for a month I3C was shown to effectively inhibit WWP1 activity, contributing to substantially inhibiting the tumor 33. Plasma concentrations of I3C reached a steady state and maintained a steady plasma concentration over almost two weeks, and the treated mice were healthy without apparent clinical symptoms 33. We observed that I3C (20 mg/kg) pre-treatment for a month alleviated myocardial inflammation by inhibiting WWP1 activity, manifested by up-regulated protein level of KLF15 and obviously suppressed inflammatory signaling pathways. Subsequently, targeting WWP1 by I3C attenuated MI-induced cardiac dysfunction and ventricular remodeling. When I3C was employed to treat recurrent respiratory papillomatosis, patients had a partial response and required surgery less often during a follow-up period 45. Because of well-established safety and pharmacokinetic profiles, I3C appears promising as a new indication of ischemic cardiovascular diseases.

The study also has limitations. We found that the cardiac phenotype was slightly better in the I3C-treated group than in the I3C-WWP1-overexpression group under the ischemic condition (Figure 10). I3C pre-treatment could rescue KLF15 degradation by overexpressed WWP1, but this reversion did not produce KLF15 protein levels comparable to those seen after I3C pre-treatment alone in the hearts of MI-mice. This observation indicated that a 20 mg/kg I3C dose might not completely inhibit the excess WWP1 generated by AAV9. Interestingly, the data presented in our study supported that overexpression of WWP1 promotes ubiquitination and degradation of KLF15 in hypoxia, but not under normal conditions. The ubiquitination of protein substrates is reported to, in part, depend on E3 protein expression or modulated by phosphorylation through interaction with adaptor proteins 46. Here, hypoxia induced post-translational modifications of KLF15, which may activate regulatory factors in the internal microenvironment, enhancing the E3 ligase activity of WWP1 or making KLF15 a better substrate for WWP1. However, we have not explored the target protein association/disassociation with KLF15 or WWP1 that could possibly change the affinity of WWP1 for KLF15. Furthermore, sex is a key risk factor for human cardiovascular diseases, but male mice are usually used in basic research 47. Women have lower rates of ischemic injury than age-matched men, but the incidence in women increases after menopause 48. Studies have demonstrated that the XX genotype resulted in larger infarct size and lower recovery after reperfusion 49. Mice and humans exhibit strong similarities in the fundamental determinants of sex. The results in the present study using male mice cannot exclude the possibility that WWP1 functions and/or mechanisms are sex dependent.

In summary, our findings demonstrated a novel role of WWP1 in myocardial ischemic injury and uncovered the mechanism underlying cardiomyocyte inflammatory response, and providing a therapeutic approach for the treating ischemic cardiomyopathy.

## Supplementary Material

Supplementary materials and methods, figures, and table.

## References

[1] Usui S, Chikata A, Takatori O, Takashima SI, Inoue O, Kato T (2019). Endogenous muscle atrophy F-box is involved in the development of cardiac rupture after myocardial infarction. J Mol Cell Cardiol.

[2] de Lemos JA, Newby LK, Mills NL (2019). A Proposal for Modest Revision of the Definition of Type 1 and Type 2 Myocardial Infarction. Circulation.

[3] Willis MS, Bevilacqua A, Pulinilkunnil T, Kienesberger P, Tannu M, Patterson C (2014). The role of ubiquitin ligases in cardiac disease. J Mol Cell Cardiol.

[4] Lu X, He Y, Tang C, Wang X, Que L, Zhu G (2020). Triad3A attenuates pathological cardiac hypertrophy involving the augmentation of ubiquitination-mediated degradation of TLR4 and TLR9. Basic Res Cardiol.

[5] Willis MS, Patterson C (2006). Into the heart: the emerging role of the ubiquitin-proteasome system. J Mol Cell Cardiol.

[6] Yu X, Kem DC (2010). Proteasome inhibition during myocardial infarction. Cardiovasc Res.

[7] Kishikawa T, Higuchi H, Wang L, Panch N, Maymi V, Best S (2021). WWP1 inactivation enhances efficacy of PI3K inhibitors while suppressing their toxicities in breast cancer models. J Clin Invest.

[8] Qin H, Pu HX, Li M, Ahmed S, Song J (2008). Identification and structural mechanism for a novel interaction between a ubiquitin ligase WWP1 and Nogo-A, a key inhibitor for central nervous system regeneration. Biochemistry.

[9] Zhi X, Chen C (2012). WWP1: a versatile ubiquitin E3 ligase in signaling and diseases. Cell Mol Life Sci.

[10] Li M, Sun G, Wang P, Wang W, Cao K, Song C (2022). Research progress of Nedd4L in cardiovascular diseases. Cell Death Discov.

[11] Basheer WA, Harris BS, Mentrup HL, Abreha M, Thames EL, Lea JB (2015). Cardiomyocyte-specific overexpression of the ubiquitin ligase Wwp1 contributes to reduction in Connexin 43 and arrhythmogenesis. J Mol Cell Cardiol.

[12] Snyder LB, Lai Y, Doviak H, Freeburg LA, Laney VK, Moore A (2021). Ubiquitin ligase Wwp1 gene deletion attenuates diastolic dysfunction in pressure-overload hypertrophy. Am J Physiol Heart Circ Physiol.

[13] Zhong G, Zhao D, Li J, Liu Z, Pan J, Yuan X (2021). WWP1 Deficiency Alleviates Cardiac Remodeling Induced by Simulated Microgravity. Front Cell Dev Biol.

[14] Kolpakov MA, Guo X, Rafiq K, Vlasenko L, Hooshdaran B, Seqqat R (2020). Loss of Protease-Activated Receptor 4 Prevents Inflammation Resolution and Predisposes the Heart to Cardiac Rupture After Myocardial Infarction. Circulation.

[15] Zhuang R, Meng Q, Ma X, Shi S, Gong S, Liu J (2022). CD4(+)FoxP3(+)CD73(+) regulatory T cell promotes cardiac healing post-myocardial infarction. Theranostics.

[16] Wang Y, Liu J, Kong Q, Cheng H, Tu F, Yu P (2019). Cardiomyocyte-specific deficiency of HSPB1 worsens cardiac dysfunction by activating NFkappaB-mediated leucocyte recruitment after myocardial infarction. Cardiovasc Res.

[17] Lin XW, Xu WC, Luo JG, Guo XJ, Sun T, Zhao XL (2013). WW domain containing E3 ubiquitin protein ligase 1 (WWP1) negatively regulates TLR4-mediated TNF-alpha and IL-6 production by proteasomal degradation of TNF receptor associated factor 6 (TRAF6). PLoS One.

[18] Tucker WO, Kinghorn AB, Fraser LA, Cheung YW, Tanner JA (2018). Selection and Characterization of a DNA Aptamer Specifically Targeting Human HECT Ubiquitin Ligase WWP1. Int J Mol Sci.

[19] Garcia-Caballero A, Gadotti VM, Stemkowski P, Weiss N, Souza IA, Hodgkinson V (2014). The deubiquitinating enzyme USP5 modulates neuropathic and inflammatory pain by enhancing Cav3.2 channel activity. Neuron.

[20] Zhao D, Zhong G, Li J, Pan J, Zhao Y, Song H (2021). Targeting E3 Ubiquitin Ligase WWP1 Prevents Cardiac Hypertrophy Through Destabilizing DVL2 via Inhibition of K27-Linked Ubiquitination. Circulation.

[21] Shao D, Villet O, Zhang Z, Choi SW, Yan J, Ritterhoff J (2018). Glucose promotes cell growth by suppressing branched-chain amino acid degradation. Nat Commun.

[22] Lu Y, Zhang L, Liao X, Sangwung P, Prosdocimo DA, Zhou G (2013). Kruppel-like factor 15 is critical for vascular inflammation. J Clin Invest.

[23] Yao Y, Li F, Zhang M, Jin L, Xie P, Liu D (2022). Targeting CaMKII-delta9 Ameliorates Cardiac Ischemia/Reperfusion Injury by Inhibiting Myocardial Inflammation. Circ Res.

[24] Wang B, Xu H, Kong J, Liu D, Qin W, Bai W (2021). Kruppel-Like Factor 15 Reduces Ischemia-Induced Apoptosis Involving Regulation of p38/MAPK Signaling. Hum Gene Ther.

[25] Hirata Y, Nomura K, Senga Y, Okada Y, Kobayashi K, Okamoto S (2019). Hyperglycemia induces skeletal muscle atrophy via a WWP1/KLF15 axis. JCI Insight.

[26] Huang CK, Dai D, Xie H, Zhu Z, Hu J, Su M (2020). Lgr4 Governs a Pro-Inflammatory Program in Macrophages to Antagonize Post-Infarction Cardiac Repair. Circ Res.

[27] Zheng H, Huang S, Wei G, Sun Y, Li C, Si X (2022). CircRNA Samd4 induces cardiac repair after myocardial infarction by blocking mitochondria-derived ROS output. Mol Ther.

[28] Chen E, Chen C, Niu Z, Gan L, Wang Q, Li M (2020). Poly(I:C) preconditioning protects the heart against myocardial ischemia/reperfusion injury through TLR3/PI3K/Akt-dependent pathway. Signal Transduct Target Ther.

[29] Feng G, Bajpai G, Ma P, Koenig A, Bredemeyer A, Lokshina I (2022). CCL17 Aggravates Myocardial Injury by Suppressing Recruitment of Regulatory T Cells. Circulation.

[30] Fan Q, Tao R, Zhang H, Xie H, Lu L, Wang T (2019). Dectin-1 Contributes to Myocardial Ischemia/Reperfusion Injury by Regulating Macrophage Polarization and Neutrophil Infiltration. Circulation.

[31] He S, Lu Y, Guo Y, Li S, Lu X, Shao S (2021). Kruppel-Like Factor 15 Modulates CXCL1/CXCR2 Signaling-Mediated Inflammatory Response Contributing to Angiotensin II-Induced Cardiac Remodeling. Front Cell Dev Biol.

[32] Huang B, Yang XD, Lamb A, Chen LF (2010). Posttranslational modifications of NF-kappaB: another layer of regulation for NF-kappaB signaling pathway. Cell Signal.

[33] Lee YR, Chen M, Lee JD, Zhang J, Lin SY, Fu TM (2019). Reactivation of PTEN tumor suppressor for cancer treatment through inhibition of a MYC-WWP1 inhibitory pathway. Science.

[34] Tao H, Zhang M, Yang JJ, Shi KH (2018). MicroRNA-21 via Dysregulation of WW Domain-Containing Protein 1 Regulate Atrial Fibrosis in Atrial Fibrillation. Heart Lung Circ.

[35] Gwechenberger M, Mendoza LH, Youker KA, Frangogiannis NG, Smith CW, Michael LH (1999). Cardiac myocytes produce interleukin-6 in culture and in viable border zone of reperfused infarctions. Circulation.

[36] Suetomi T, Willeford A, Brand CS, Cho Y, Ross RS, Miyamoto S (2018). Inflammation and NLRP3 Inflammasome Activation Initiated in Response to Pressure Overload by Ca(2+)/Calmodulin-Dependent Protein Kinase II delta Signaling in Cardiomyocytes Are Essential for Adverse Cardiac Remodeling. Circulation.

[37] El-Naccache DW, Chen F, Palma MJ, Lemenze A, Fischer MA, Wu W (2022). Adenosine metabolized from extracellular ATP promotes type 2 immunity through triggering A2BAR signaling in intestinal epithelial cells. Cell Rep.

[38] Marchant DJ, Boyd JH, Lin DC, Granville DJ, Garmaroudi FS, McManus BM (2012). Inflammation in myocardial diseases. Circ Res.

[39] Ambrosini S, Montecucco F, Kolijn D, Pedicino D, Akhmedov A, Mohammed SA (2022). Methylation of the Hippo effector YAP by the methyltransferase SETD7 drives myocardial ischemic injury: a translational study. Cardiovasc Res.

[40] Li L, Li H, Tien CL, Jain MK, Zhang L (2020). Kruppel-Like Factor 15 Regulates the Circadian Susceptibility to Ischemia Reperfusion Injury in the Heart. Circulation.

[41] Napoli C, Benincasa G, Donatelli F, Ambrosio G (2020). Precision medicine in distinct heart failure phenotypes: Focus on clinical epigenetics. Am Heart J.

[42] Zhao Y, Song W, Wang L, Rane MJ, Han F, Cai L (2019). Multiple roles of KLF15 in the heart: Underlying mechanisms and therapeutic implications. J Mol Cell Cardiol.

[43] Zhang Q, Wang L, Wang S, Cheng H, Xu L, Pei G (2022). Signaling pathways and targeted therapy for myocardial infarction. Signal Transduct Target Ther.

[44] Gong R, Jiang Z, Zagidullin N, Liu T, Cai B (2021). Regulation of cardiomyocyte fate plasticity: a key strategy for cardiac regeneration. Signal Transduct Target Ther.

[45] Anderton MJ, Manson MM, Verschoyle RD, Gescher A, Lamb JH, Farmer PB (2004). Pharmacokinetics and tissue disposition of indole-3-carbinol and its acid condensation products after oral administration to mice. Clin Cancer Res.

[46] Yang LL, Xiao WC, Li H, Hao ZY, Liu GZ, Zhang DH (2022). E3 ubiquitin ligase RNF5 attenuates pathological cardiac hypertrophy through STING. Cell Death Dis.

[47] Reue K, Wiese CB (2022). Illuminating the Mechanisms Underlying Sex Differences in Cardiovascular Disease. Circ Res.

[48] Ostadal B, Ostadal P (2014). Sex-based differences in cardiac ischaemic injury and protection: therapeutic implications. Br J Pharmacol.

[49] Murphy E, Steenbergen C (2007). Gender-based differences in mechanisms of protection in myocardial ischemia-reperfusion injury. Cardiovasc Res.

