# Supplementary material for: Targeting WWP1 ameliorates cardiac ischemic injury by suppressing KLF15-ubiquitination mediated myocardial inflammation
Source: Theranostics. 2023 Jan 1;13(1):417–37. doi: 10.7150/thno.77694 (PMC9800727; doi:10.7150/thno.77694)
Supplement: Supplementary file 1 — Supplementary materials and methods, figures, and table. [file thnov13p0417s1.pdf]

SUPPLEMENTARY MATERIALS

Targeting WWP1 ameliorates cardiac ischemic injury by suppressing KLF15-ubiquitination mediated myocardial inflammation

Xia Lu[1#], Boshen Yang[1#], Ruiqiang Qi[2], Qifei Xie[2], Taixi Li[1], Jie Yang[3], Tingting Tong[3], Kaifan Niu[1], mingyu Li[4], Weijun Pan[5], Yongxin Zhang[6], Dongmei Shi[1], Suiji Li[2], Cuilian Dai[2], Chengxing Shen[1], Xiaoqing Wang[1]*, Yan Wang[2]* and Juan Song[2]*

[1]Department of Cardiology, Shanghai Sixth People's Hospital Affiliated to Shanghai Jiao Tong University School of Medicine, Shanghai 200233, China.

[2] Xiamen Cardiovascular Hospital, Xiamen University, Xiamen 361004, China.

[3] Key Laboratory of Targeted Intervention of Cardiovascular Disease, Collaborative Innovation Center for Cardiovascular Disease Translational Medicine, Nanjing Medical University, Nanjing 211166, Jiangsu, China.

[4]Fujian Provincial Key Laboratory of Innovative Drug Target Research, School of Pharmaceutical Sciences, Xiamen University, Xiamen 361102, China.

[5]Key Laboratory of Tissue Microenvironment and Tumor, CAS Center for Excellence in Molecular Cell Science, Shanghai Institute of Nutrition and Health, Shanghai Institutes for Biological Sciences, University of Chinese Academy of Sciences, Chinese Academy of Sciences (CAS), Shanghai, China.

[6] The first clinical medical college, Southern Medical University, Guangzhou 510000, China.

*Corresponding authors:

Juan Song: songjuan_ok@163.com

Yan Wang: wy@medmail.com.cn

Xiaoqing Wang: xq_w@outlook.com

[#]These authors contributed equally to this work.

MATERIALS AND METHODS

Echocardiography

Cardiac functions were evaluated by echocardiography (GE Vivid 7 equipped with a 14-MHz phase array linear transducer, S12, allowing a 150 maximal sweep rate) in Figure 2, Figure 8, Figure 11, and Figure S5. Cardiac functions were tested with a high-resolution ultrasound imaging system (MyLab Touch: Esaote, Italy linear array probe, frequency 18-22 MHz) in Figure 7. All measurements were performed by an observer blinded to the identities of the tracings and averaged over five consecutive cardiac cycles. Left ventricle (LV) end diastolic volume (LVEDV) and LV end systolic volume (LVESV) were calculated using the biplane area-length method. LV ejection fraction (EF%) was calculated following the formula: EF% = [(LVEDV-LVESV)/LVEDV] × 100%. The 2D-guided left ventricular M mode tracings at the papillary muscle level were recorded from the long-axis view to measure LV internal diameter at end systole (LVIDs) and at end diastole (LVIDd). LV fractional shortening (FS%) was calculated according to the following formula: FS% = [(LVIDd-LVIDs)/LVIDd] × 100%. The representative image of each group was selected based upon the mean value.

Cell culture

Neonatal rat cardiac myocytes (NRCMs) were isolated from 1 to 3-day-old neonatal Sprague-Dawley rats (Beijing Vital River Laboratory Animal Technology Co., Ltd.). NRCMs were plated at a density of 1×10^6 cells/ml and cultured in Dulbecco’s Modified Eagle Medium (DMEM, Invitrogen Corporation, USA), supplemented with penicillin, streptomycin, and 10% FBS. The cells were put on serum-free medium and subsequently infected with Adv-GFP (MOI = 50) or Adv-WWP1 (MOI = 50) for 4 h, and then the medium containing the adenovirus was removed and replaced by DMEM supplemented with 10% FBS. After 48 h of infection, NRCMs cultured in serum-free and glucose deprived DMEM under hypoxia for 6 h induced by a hypoxia chamber (Thermo, HERA cell 150i) with 5% CO_2, 1% O_2, and 94% N_2. NRCMs cultured in serum-free medium and normoxia with 5% CO_2 acted as controls. H9C2 cells and RAW264 cells purchased from Cell Bank of the Chinese Academy of Sciences (Shanghai, China) were cultured in DMEM supplemented with 10% FBS, penicillin and streptomycin. H9C2 cells were seeded into 96-well plates. Cell Counting Kit-8 (CCK-8) (DOJINDO, Japan, Cat#AY-4710P) were used to analysis the cell viability according to the instruction.

Western blot

Briefly, Western blot analyses were performed using commercially available antibodies: anti-WWP1 (ab43791, abcam, 1:1000), anti-KLF15 (sc-271675, Santa Cruz, 1:100), anti-Ub K48 (12805S, abcam, 1:1000), anti-Bcl2 (15071S, SCT, 1:1000), anti-Bax (5023S, CST, 1:1000), anti-Cleaved-caspase 3 (9661S, CST, 1:1000), anti-p65 (AF1234, Beyotime, 1:1000), anti-p300 (86377S, CST, 1:1000), anti-AcK310 (19870, abcam, 1:1000), anti-P38 (AF1111, Beyotime, 1:1000), anti-ERK1/2 (AF1051, Beyotime, 1:1000), anti-p-ERK1/2 (AF1891, Beyotime, 1:1000), anti-GAPDH (HRP60004, Proteintech, 1:10000), anti-Tubulin (AF7819-1, Beyotime, 1:1000), anti-LaminB1 (AF1408, Beyotime, 1:1000), anti-CD68 (A6554, Abclonal, 1:1000), anti-Ly6G (A20861, Abclonal, 1:1000), anti-Flag (AF519-1, Beyotime, 1:1000), anti-HA (AF5057, Beyotime, 1:1000) followed by incubation with peroxidase-conjugated secondary antibodies. The signals were detected with the enhanced-chemiluminescent (ECL) system (Tanon, China) and quantified by scanning densitometry with the Image J software. GAPDH and Lamin B1 served as the loading control. The results from each experimental group were expressed as relative integrated intensity compared with the control group measured at the same time.

Real-time PCR

Table S1. Primer sequences.

Genes	Primer sequences	
Mus HPRT	Forward	GTTGGATACAGGCCAGACTTTGTT
	Reverse	GATTCAACTTGCGCTCATCTTAGGC
Mus IL-6	Forward	CCGGAGAGGAGACTTCACAG
	Reverse	TCCACGATTTCCCAGAGAAC
Mus IL-1β	Forward	GCAACTGTTCCTGAACTCAACT
	Reverse	ATCTTTTGGGGTCCGTCAACT
Mus VCAM-1	Forward	GAACCCAAACAGAGGCAGAG
	Reverse	GGTATC CCATCACTTGAGCAG
Mus MCP-1	Forward	TAGCATCCACGTGCTGTCTC

	Reverse	CAGCCGACTCATTGGGATCA
Rat HPRT	Forward	GCTGAAGATTTGGAAAAGGTGT
	Reverse	ACAGAGGGCCACAATGTGAT
Rat IL-6	Forward	ACCAGAGGAAATTTTCAATAGGC
	Reverse	TGATGCACTTGCAGAAAACA
Rat IL-1β	Forward	GGTCAAAGGTTTGGAAGCAG
	Reverse	TGTGAAATGCCACCTTTTGA
Rat TNF-α	Forward	AGGGTCTGGGCCATAGAACT
	Reverse	CCACCACGCTCTTCTGTCTAC

TUNEL assay

The heart tissues were embedded in paraffin, cut at 8 μm, dewaxed and rehydrated, and then permeabilized with 20 μg/mL proteinase K for 10 min. The staining was performed using an In Situ Cell Death Detection Kit (12156792910, Roche) according to the protocol. Positive controls were administered with DNase I, while TdT was omitted from the reaction process to provide negative controls. The percentage of apoptotic cardiomyocytes was calculated as TUNEL-positive nuclei number with simultaneous cTnI-positive staining divided by the total number of cTnI-positive stained cells. Five random fields of vision were photographed with a confocal microscope (Zeiss, German). For cultured cells, NRCMs were fixed with formaldehyde for 15 min on ice and washed with PBS. NRCMs were permeabilized with 0.3% Triton X-100 for 5 min. TUNEL detection solution was added and incubated with NRCMs at 37 ℃ for 60 min away from light. NRCMs were washed with PBS and stained with DAPI sequentially. Then, five fields were randomly selected to take pictures in each group with fluorescence microscope (OLYMPUS TH4-200, Japan). TUNEL-positive nuclei numbers were quantified by Image J software.

SUPPLEMENTARY RESULTS

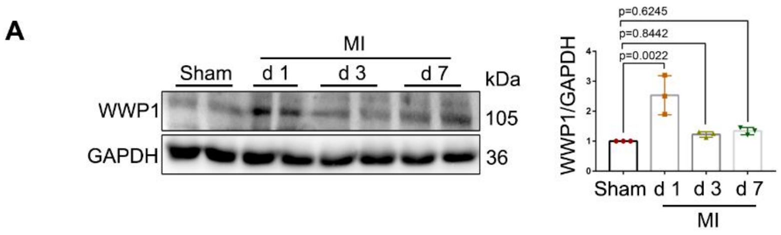

Figure S1. WWP1 expression peaks on the first day post-MI. A Mice hearts subjected to LAD ligation were harvested at day 1, 3, and 7, respectively. Representative Western blot was performed to detect the temporal protein expression pattern of WWP1 in the infarct area (infarct and border zone), and statistical result was shown. n = 3. The data are shown as the means ± SD. The data shown was analysed by unpaired Student's t-test.

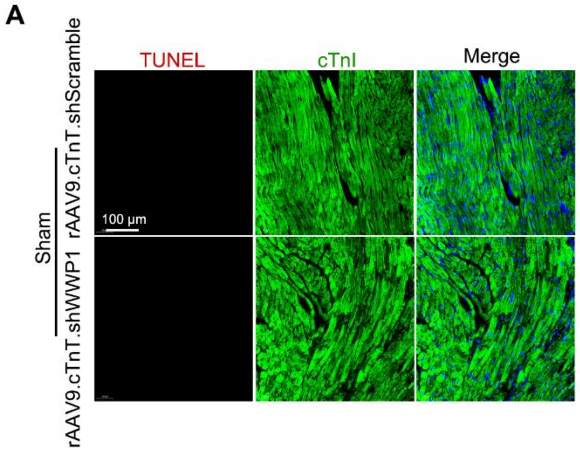

Figure S2. Cardiomyocyte-specific overexpressing WWP1 does not affect the apoptosis of cardiomyocytes in sham-mice hearts. A Mice were treated with rAAV9-cTnT-shScramble or rAAV9-cTnT-shWWP1 by intravenous injection of tail two weeks before suffering from sham or LAD ligation, and after additional 3 days, mice were sacrificed. TUNEL assay and immunofluorescence staining with cTnI were performed to detect cardiomyocyte apoptosis in the myocardium. Scale bar = 100 μm. n = 3.

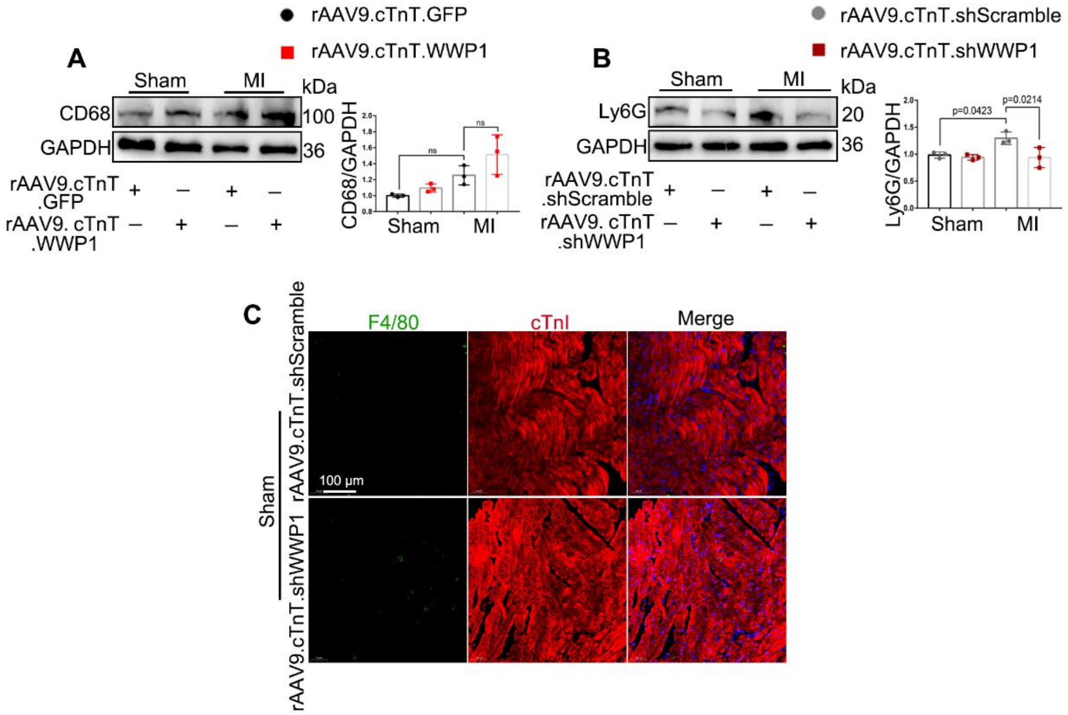

Figure S3. WWP1 affects the inflammation of cardiomyocytes at early phase post-MI. A, B Representative Western blots were performed to detect the CD68 and Ly6G expression in the infarct area, and statistical results were shown. n = 3. **C** Immunofluorescence co-staining for cTnI with F4/80 and DAPI in infarcted hearts. Scale bar = 100 μm. n = 3. The data are shown as the means ± SD. The data shown in **A**, and **B** were analysed by one-way ANOVA followed by Bonferroni post hoc test.

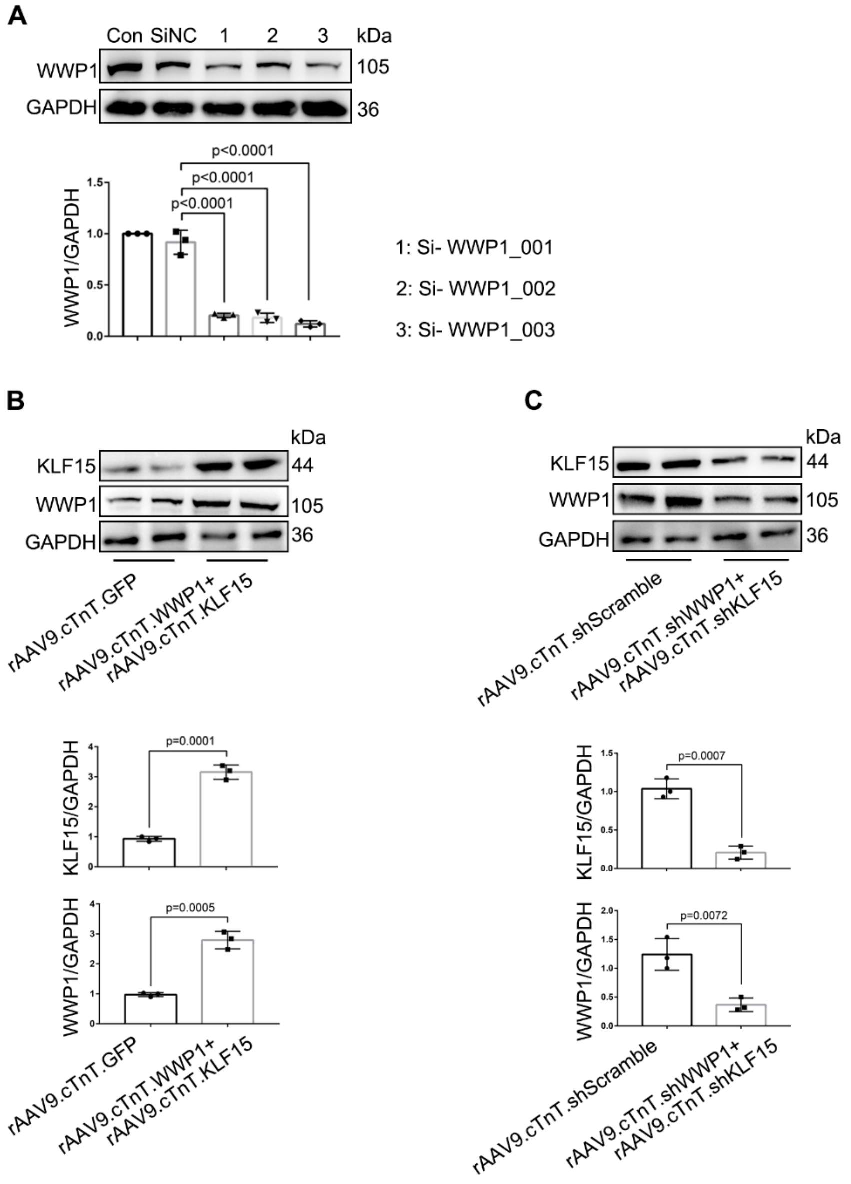

Figure S4. The protein expression of WWP1. A H9C2 cells were transfected with Si-WWP1 for 36 h, and representative Western blot was performed to test the protein change of WWP1. n = 3. **B** Mice were injected with rAAV9-cTnT-WWP1 with or without rAAV9-cTnT-KLF15 for two weeks, and representative Western blot was performed to test the protein change of WWP1 in mice hearts. n = 3. **C** Mice were injected with rAAV9-cTnT-shWWP1 with or without rAAV9-cTnT-shKLF15 for two weeks, and representative Western blot was performed to test the protein change of WWP1 in mice hearts. n = 3. The data are shown as the means ± SD. The data shown in **A** was analysed by one-way ANOVA followed by Bonferroni post hoc test, and shown in **B**, and **C** were analysed by unpaired Student’s t-test.

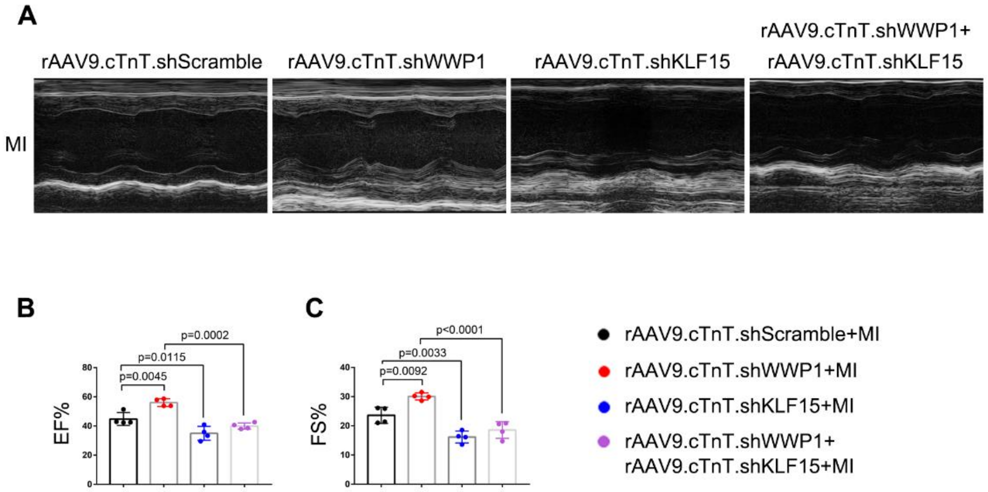

Figure S5. The regulatory role of WWP1 on cardiac function post-MI is dependent on KLF15. Mice were injected with rAAV9-cTnT-shWWP1 with or without rAAV9-cTnT-shKLF15 before they subjected to MI. **A-C** Cardiac function were measured by echocardiography at day 3 post-MI. n = 4. The data are shown as the means ± SD. The data shown in **B,** and **C** were analysed by one-way ANOVA followed by Bonferroni post hoc test.

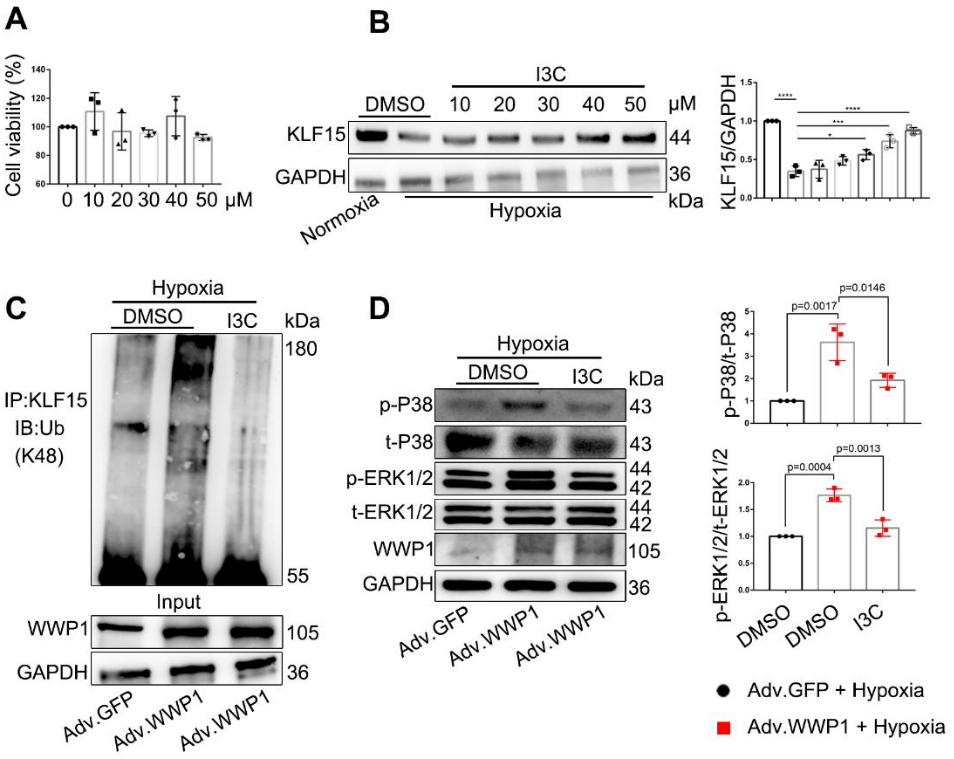

Figure S6. I3C treatment suppresses KLF15-degradation mediated activation signals in hypoxia-induced H9C2 cells. A H9C2 cells were infected with Adv-WWP1 or Adv-GFP for 24 h followed by I3C (50 μM) treatment for 24 h, and then the cells were treated with hypoxia for 6 h. CCK8 was applied to detect the viability of H9C2 cells treated with different concentration of I3C. n = 3. **B** Representative Western blot was performed to test the protein change of KLF15 in hypoxia-induced H9C2 cells treated with different concentration of I3C. n = 3. **C** Cellular proteins were isolated for immunoprecipitation with anti-KLF15 antibody followed by immunoblot with anti-Ub-K48 antibody. n = 3. **D** Levels of phosphorylated P38 and ERK1/2 were examined by Western blots, and statistical results were shown. n = 3. The data are shown as the means ± SD. The data shown in **A**, **B**, and **D** were analysed by one-way ANOVA followed by Bonferroni post hoc test.

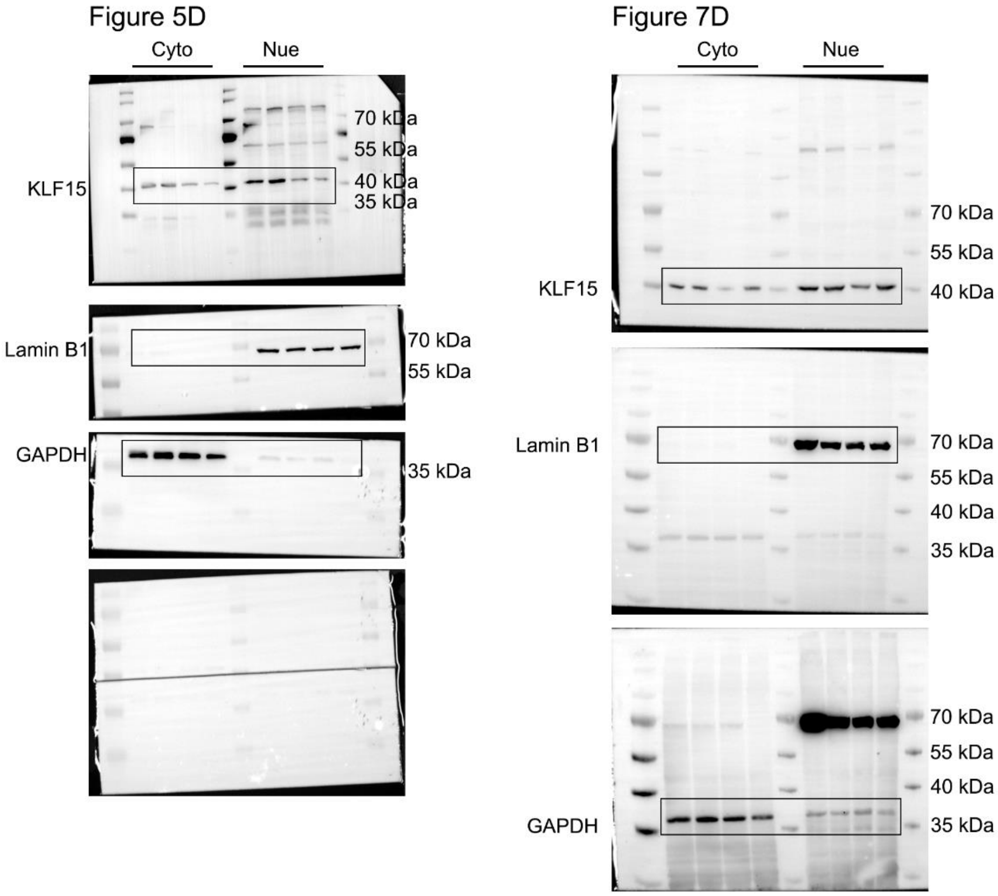

Figure S7. Images of Western blot without cutting.
